# Comparative analysis of *Erycibe schmidtii* Craib and its potential substitutes based on metabolites and pharmacodynamic effect

**DOI:** 10.3389/fphar.2025.1510170

**Published:** 2025-05-12

**Authors:** Ning Li, Chen-Yu Ye, Jing Hu, Tong Qu, Wen-Jing Lu, Xiao-Min Cui, Chao Liang, Zhi-Yong Chen, Hui Ren, Chang-Jiang-Sheng Lai

**Affiliations:** ^1^ Laboratory of Chinese Medicine Chemistry, Institute of Traditional Chinese Medicine, Shaanxi Academy of Traditional Chinese Medicine, Xi’an, Shaanxi, China; ^2^ College of Life Sciences, Northwest University, Xi’an, Shaanxi, China; ^3^ Institute of Collaborative Innovation in Great Health, College of Biotechnology and Food Science, Tianjin Key Laboratory of Food Biotechnology, Tianjin University of Commerce, Tianjin, China

**Keywords:** Erycibe schmidtii Craib, Porana sinensis Hemsl., potential substitutes, LC-MS/MS, rheumatoid arthritis, pharmacodynamic effect

## Abstract

**Background:**

*Erycibe schmidtii* Craib (*Esc*), a traditional treatment for rheumatoid arthritis (RA), faces resource scarcity, leading to the emergence of potential substitutes in the market. Although these potential substitutes have shown properties that alleviate RA-symptoms, their therapeutic equivalence to *Esc* still requires systematic validation.

**Purpose:**

This study aims to identify suitable potential substitutes for *Esc* and elucidate their therapeutic mechanisms for RA by conducting comparative analyses of metabolites and pharmacology among these potential substitutes.

**Methods:**

Six botanical samples were analyzed *via* LC-MS/MS for metabolite profiling and phenolic quantification. Pharmacological comparisons employed LPS-stimulated RAW264.7/MC3T3-E1 and MH7A cell models. Mechanistic studies on macrophage polarization (LPS/IL-4-induced RAW264.7), osteoblast mineralization, and synoviocyte behaviors (proliferation/migration/invasion) were conducted for top candidates.

**Results:**

A total of 54 metabolites were identified in the samples by LC-MS/MS. *Pse* showed the highest metabolite similarity to *Esc*, and both *Pse* and *Psh.*V contained higher levels of phenolic compounds than *Esc*. Combined with the pharmacodynamic results, *Pse* was superior *Psh.*V in anti-RA efficacy and was the only comparable potential substitute. Mechanistically, both *Esc* and *Pse*: Modulated M1/M2 macrophage polarization; Enhanced osteogenic markers (Runx2, Osx, Ocn) and mineralization; Inhibited synoviocyte proliferation/migration/invasion via Bcl-2 suppression and Caspase-3 activation.

**Conclusion:**

Multidimensional analysis confirmed that *Pse* is the optimal potential substitute for *Esc*, with high similarity in both metabolites and biological activities between the two. Both botanical medicines can slow the progression of RA by regulating immune responses, stimulating osteoblast differentiation, and inducing synoviocyte apoptosis. This study provides critical evidence for the sustainable utilization of *Esc* resources and expands treatment options for RA.

## 1 Introduction

Rheumatoid arthritis (RA) is a chronic autoimmune disease characterized by joint lesions, and it may occur in individuals at any age, and the global prevalence of RA ranges from 0.5% to 1% (Ge et al., 2022). The etiology of RA is complicated and affected by various factors, including genetics, environmental factors, and immunological abnormalities; however, it remains undefined about its etiology (Deng et al., 2023). The pathological characteristics of RA mainly include the changes in persistent synovitis, the formation of hyperplastic synovial pannus tissues, and the destruction of cartilage and bones (Chen et al., 2023), which can damage both joints and extra-articular organs, including the heart, kidney, lung, digestive system, eyes, skins, and nervous system (Radu and Bungau, 2021). Modern pathological studies have revealed that synovial hyperplasia and inflammation are central to rheumatoid arthritis (RA) pathogenesis, serving as the pathological foundation for secondary lesions such as bone and cartilage destruction. Fibroblast-like synoviocytes (FLSs) play a pivotal role in this process by secreting pro-inflammatory cytokines (e.g., TNF-α, IL-6) and matrix metalloproteinases (MMPs), driving synovial inflammation and cartilage degradation. Following synovial inflammation in RA, bone destruction occurs through a two-step pathological mechanism: osteoclast-mediated bone resorption triggered by RANKL (receptor activator of nuclear factor κ-B ligand) secreted from FLSs and immune cells, leading to excessive degradation of bone matrix via acidification and protease activity; and impaired osteoblast function caused by inflammatory cytokines (e.g., IL-17), which disrupt osteoblast differentiation and activity resulting in insufficient bone formation. The imbalance between osteoclastic resorption and osteoblastic formation ultimately leads to net bone loss (Zhang et al., 2022). Currently, the key therapeutic strategies for RA are mainly implemented based on drugs and surgical procedures, including disease-modifying anti-rheumatic drugs, non-steroidal anti-inflammatory drugs, glucocorticoids, and biological response modifiers. Although these drugs may alleviate synovitis and systemic inflammation (Li et al., 2022), long-term disease remission may not be achieved due to the adverse effects of these drugs, including gastrointestinal reactions, aberrant changes in liver functions, and bone marrow inhibition. Therefore, there is an urgent demand for new approaches to the effective treatment of RA (Ge et al., 2022).

Traditional Chinese medicines (TCMs) have attracted wide attention owing to their multi-compound and multi-target properties. Compared with those drugs with a requirement for pharmaceutical synthesis, TCMs present higher availability and safety and lower costs. As per the 2020 edition of the *Chinese Pharmacopoeia*, *Dinggongteng* is defined as the dried stems of *Erycibe obtusifolia* Benth (*Eob*) or *Erycibe schmidtii* Craib (*Esc*) from the Convolvulaceae family. This botanical drug exhibits significant therapeutic effects in treating RA (Commission, 2020; Fan et al., 2021). As a widely used traditional medicinal plant, *Esc* possesses diverse bioactive metabolites and demonstrates significant clinical application potential. Previous studies have identified that it primarily contains chemical metabolites including flavonoids, chlorogenic acid derivatives, coumarins, alkaloids, and esterified glycosides (Xue, 2020). Besides, modern pharmacological research has also proved that it has analgesic, anti-inflammatory, and pupil constriction effects, and it can lower intraocular pressure and improve respiratory immune functions (Hu et al., 2020). Chen et al. (2013) investigated the anti-inflammatory effects of 40% ethanolic extracts from *Eob* and *Esc* using xylene-induced mouse ear edema, formaldehyde-evoked inflammatory response, and carrageenan-induced air pouch synovitis models. Results demonstrated that oral administration of *Eob* extract (480 mg/kg) and *Esc* (612 mg/kg) inhibited ear edema by 34.6% and 39.4%, respectively, and suppressed formaldehyde-induced inflammation by 23.3% and 28.1%. Both extracts significantly inhibited prostaglandin E2 (PGE2) synthesis in the carrageenan-induced synovitis model, with acute toxicity assays confirming high oral safety. Zeng et al. (1999) pharmacologically validated that erycibe alkaloid II (a muscarinic receptor-specific agonist) induces dual effects of pupillary constriction and intraocular pressure modulation in rabbit ocular models, mediated by selective activation of the M3 receptor signaling pathway. Pan et al. (2011) demonstrated through *in vitro* assays that *Eob* exhibits potent scavenging activity against DPPH and ABTS free radicals, alongside significant inhibition of lipid peroxidation in murine hepatic, splenic, and renal tissues. At present, more than 10 kinds of Chinese patent medicines have been developed with *Esc* as the main drug, including FengShiDieDaYaoJiu (FengLiaoXing), TengLuoNingJiaoNang, and GuTongTieGao. These drugs play an important role in the treatment of RA, injuries from falls and other diseases (Liu et al., 2020; Qu et al., 2022a; Qu et al., 2022b). However, in recent years, with the deterioration of the ecological environment and human excessive mining, the wild medicinal resources of *Eob* and *Esc* have been nearly exhausted. Currently, there is only *Esc* available on the market, which cannot meet the daily medicinal demands. The deficiency in plant resources results in many substituents of *Esc*, seriously affecting medical safety.

To guarantee the clinical efficacy and safety application of *Esc* and its formulas and promote the sustainable utilization of the plant resources of *Esc*, it is necessary to carry out systematic and in-depth research on *Esc* and its substituents. If left unchecked, further unsustainable exploitation of such endemic medicinal plant species may lead to their extinction in the near future. Current advancements in biotechnology for endangered species conservation have established a multidimensional framework (1) Gene editing technologies (e.g., CRISPR-Cas9): enable targeted genome modification to enhance stress resistance, disease tolerance, and environmental adaptability; (2) Ecological restoration systems integrate microbial-assisted remediation (e.g., soil microbiome regulation) with tissue culture-based revegetation techniques to synergistically rehabilitate habitats; (3) Germplasm repositories coupled with bioinformatics allow digital preservation and intelligent analysis of genetic resources; (4) Artificial propagation protocols incorporate ecological acclimation training (e.g., simulated natural light/humidity regimes) to improve post-release survival rates; (5) Germplasm cryopreservation establishes “genetic insurance” for species continuity (Ahmar et al., 2020; Basu et al., 2018; Roque-Borda et al., 2021). While these technologies are evolving from single-species protection to holistic ecosystem intervention, they require careful balancing of innovation with ethical risks (e.g., genetic contamination, ecological cascade effects). Furthermore, high costs and unequal resource allocation hinder widespread implementation. Concurrently, development of alternatives alleviates exploitation pressure on wild populations. Future efforts should focus on fostering interdisciplinary collaborations, establishing global ethical guidelines, and designing sustainable utilization-conservation integration models.

**FIGURE 1:**
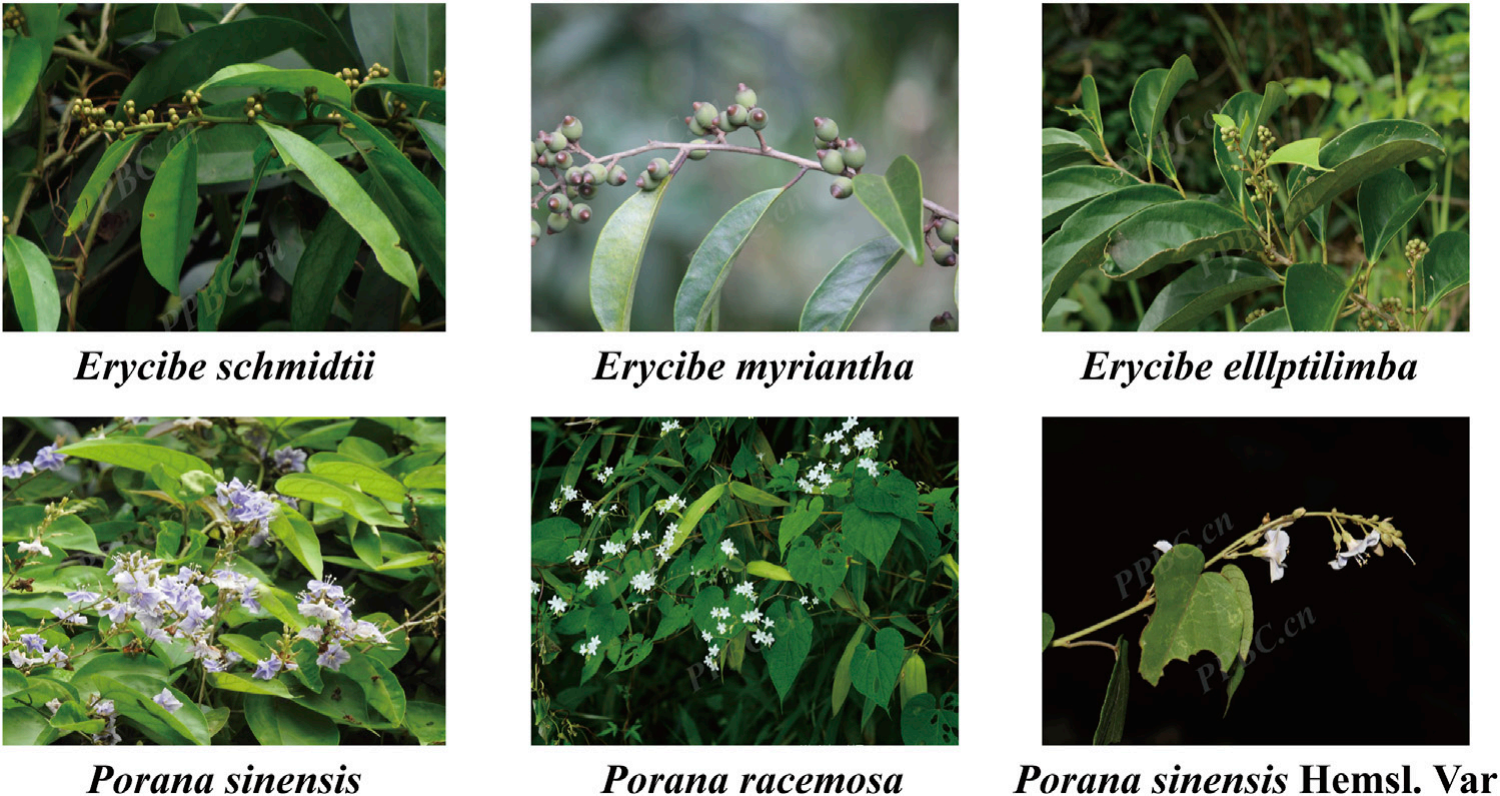
Plant morphology of *Erycibe schmidtii* and five potential substituents.

In the early stage, the research group delved into *Esc* by analyzing its chemical metabolites and pharmacological effects. The high-resolution mass spectrometry analysis results revealed that *Esc* contained coumarins, chlorogenic acid derivatives, alkaloids, and other chemical metabolites (Hu et al., 2020). The anti-inflammatory, analgesic, and acute toxicity test results demonstrated that 40% ethanol extracts of *Esc* had anti-inflammatory and analgesic effects, exhibiting high safety of oral administration (Chen et al., 2013). In addition, it has been reported that the chemical metabolites of *Esc* have anti-inflammatory effects. For example, scopolamine displayed anti-arthritic activities *in vivo* by inhibiting synovial fluid angiogenesis. Besides, scopolin also showed anti-RA activities in the adjuvant-induced arthritis (AIA) rat model. It had significant inhibitory effects on neovascularization in synovial tissues at 100 mg/kg and the ability to inhibit the expression of IL-6, VEGF, and FGF-2 in the synovium (Zhou, 2016). Meanwhile, the research group also carried out some studies on *Porana sinensis* Hemsl. (*Pse*), an potential substitute of *Esc*. It was unraveled that *Pse* did show good anti-inflammatory activities, and it had a certain effect on gout arthritis and RA (Du et al., 2020; Hu et al., 2022). To promote the sustainable development of medicinal materials and expand their application scope, this study extended its investigation to the *Erycibe* and *Porana* genera (Convolvulaceae). Through preliminary screening conducted by our research group, five potential substitutes were identified (plant morphology shown in Figure 1, image sourced from https://www.iplant.cn/). Building on this foundation, we conducted a systematic comparison of the metabolite and pharmacological activities between *Esc* and its potential substitutes. These findings may provide a scientific basis for broadening the medicinal applications of *Esc* while enhancing the safety of clinical medication.

## 2 Materials and methods

### 2.1 Materials and reagents

#### 2.1.1 Medicinal materials

*Esc* (Lot No. 20111020), *Erycibe myriantha* Merr. (Lot No. 20110918), *Erycibe eliptilimba* Merr. et Chun (Lot No. 20111023), *Pse* (Lot No. 20130717), *P. sinensis* Hemsl. var. delavayi (Gagn.et Courch.) Rehd. (Lot No. 20140825), *Porana racemosa* Roxb. (Lot No. 20201027) was cleaned, sliced, dried and preserved for spare use, which were identified by Dr. Zhiyong Chen, and deposited at the Shaanxi Academy of Traditional Chinese Medicine.

#### 2.1.2 Standard substance

Scopolin (wkq20021510), Cryptochlorogenic acid (wkq20082705), Isochlorogenic acid A (wkq20020403), Isochlorogenic acid B (wkq20021003) and Isochlorogenic acid C (wkq20031101) were purchased from the Sichuan Weikeqi Biological Technology CO., LTD (Chengdu, China); Scopoletin (161,208), Chlorogenic acid (1701904), Neochlorogenic acid (17062003), Umbelliferone (18010202), Esculetin (18092803) and caffeic acid (17122804) were bought from the Shanghai Qiming Bioengineering Institute (Shanghai, China). All reference standards used in this study had purity exceeding 98%, and their purity and chemical structures were reconfirmed by HPLC and mass spectrometry prior to use.

#### 2.1.3 Reagents

Chromatographically pure methanol (Thermo Fisher Scientific, USA), chromatographically pure formic acid (Sigma-Aldrich, USA), both of LC-MS grade, ultrapure water (Watson’s distilled water), and other reagents were analytically pure. Fetal calf serum (FBS, Gibco, J20230113), Thiazolyl blue (MTT, Shanghai McLean biochemical technology co., LTD, C10769613), LPS (Sigma, P2636866), DMEM medium (C11995500BT, Gibco, USA), Penicillin-Streptomycin Liquid (Solarbio, 20221231), DMSO (No: 1121E0316), NO Kits (Beyotime, 110922230417), IL-6 Elisa kit (EM0121); TNF-α Elisa kit (EM0183); IL-1β Elisa kit (EM0109) all purchased from Wuhan Fine Biological Technology Co., LTD. All-in-One First-Strand Synthesis MasterMix (Lablead,F0202), 2x Realab Green PCR Fast mixture (Lablead,R0202), ALP activity kit (Beyotime, P0321S), MC3T3-E1 Subclone 14 osteogenic differentiation medium (Procell, PD-033), Crystal violet staining solution (Solarbio, G1063),The following antibodies were used: Actin (Rabbit, Servicebio, GB11001), MMP-3 (Rabbit, Servicebio, GB11131), Caspse-3 (Rabbit, Servicebio, GB11767C), MMP-9 (Rabbit, Abclonal, A0289), Bcl-2 (Rabbit, Servicebio, GB154380), IgG-HRP (Servicebio, GB23303). The primers used in the RT-qPCR were synthesized by Sangon Biotech (Shanghai) Co., Ltd. (Shanghai China).

### 2.2 Methods

#### 2.2.1 Determination of phenolic metabolites

##### 2.2.1.1 Preparation of botanical drug extracts

All collected botanical drugs were numbered from S1 to S6(S1, *Erycibe schmidtii*; S2, *Erycibe myriantha*; S3, *Erycibe elllptilimba*; S4, *P. sinensis*; S5, *P. sinensis* Hemsl. Var; S6, *P. racemosa*.). Then, these botanical drugs were dried at 50°C for 24 h, crushed with a pulverizer, and sieved through a 60-mesh pharmacopoeia sieve. Subsequently, each ground botanical drug was accurately weighed (5.0 g) and transferred to a 50 mL centrifuge tube, followed by the immediate addition of 30 mL of methanol: water (80∶20 v/v). After the mixture was sealed and extracted in an ultrasonic bath for 30 min, the tube was centrifuged at 10,000 rpm for 2 min, and the supernatant was collected. These extraction procedures were repeated in triplicate. Next, the supernatants extracted from each botanical drug in triplicate were mixed, filtered, concentrated under a decreased pressure, and freeze-dried to obtain the dried extract of each botanical drug for subsequent experiments. After an appropriate amount of dried extract was weighed from each botanical drug and then dissolved in 80% methanol, the mixture was filtered through a 0.22 μm filter membrane to determine phenolic substances for the qualitative analysis of their antioxidant capacity.

##### 2.2.1.2 Total favonoids

The content of total flavonoids in the six botanical drugs (S1-S6) was determined by an aluminum chloride colorimetric assay with slight modifications (Xue et al., 2017). Firstly, a properly diluted individual solution sample (120 μL) was mixed with 8 μL of sodium nitrite (50 mg/mL) in the designated well of a 96-well microplate. Then, the microplate was stood for 6 min before the addition of 8 mL of aluminum chloride (100 mg/mL) into each well. After the incubation at room temperature for 5 min, 100 mL of sodium hydroxide (40 mg/mL) was added to each well. Subsequently, the mixture was mixed properly by pipetting up and down 10 times. Next, the microplate was covered and incubated in darkness at room temperature for 30 min, and the absorbance was measured at 410 nm by using an enzyme-labeled instrument. The experimental results were expressed as rutin equivalents in the calibration curve of the rutin standard solution (0–100 mg/L) and as rutin equivalents in every 1 g of dried extract of botanical drugs. All experiments were repeated in triplicate.

##### 2.2.1.3 Total phenols

The content of total phenols in the six botanical drugs (S1-S6) was determined using Folin-Ciocalteau’s method with slight modifications (Ma et al., 2018). Firstly, a properly diluted individual solution sample (20 μL) was mixed with 40 μL of Folin-Ciocalteau reagent (25%) in the corresponding well of a 96-well microplate. After the standing procedure at room temperature for 5 min, 140 μL of sodium carbonate solution (700 mM) was added to each well and the plate was shaken in an orbital shaker at 500 rpm for 30 s. Then, the microplate was covered and incubated in darkness at 40°C for 30 min, followed by reading at 765 nm using the enzyme-labeled instrument. According to the calibration curve of the gallic acid standard solution (0–400 mg/L), the results were expressed as gallic acid equivalents and as gallic acid equivalents in mg per 1 g of dried extracts of botanical drugs. All experiments were repeated in triplicate.

##### 2.2.1.4 Total tannins

The content of total tannins in the six botanical drugs (S1-S6) was determined based on the phosphomolybdenum tungstic acid-casein reaction described by Ma et al. (2018) with slight modifications. Firstly, a properly diluted individual solution sample was mixed with casein at a ratio of 1∶4 (ml ∶ mg) and incubated indoors at 200 rpm, followed by shaking for 3 h. Then, the mixture was filtered through a 0.45 mm filter, and the supernatant was collected. After the precipitation reaction of casein, the reactant was named as the corresponding sample. The remaining steps were the same as the method for determining the content of total phenols. The content of total tannins was equal to the difference in the content of total phenols before and after the precipitation reaction of casein. All measurements were conducted in triplicate, and the results were expressed as gallic acid equivalents according to the calibration curve of the gallic acid standard solution (0–400 mg/L) and as gallic acid equivalents in mg per 1 g of dried medicinal extracts.

#### 2.2.2 Determination of the activity of DPPH radical scavenging

In a 96-well plate, 100 μL of 0.4 mg/mL 1,1-Diphenyl-2-picryl-hydrazyl (DPPH) working solution and 100 μL of test solution were added in sequence and mixed properly, followed by the incubation at room temperature for 30 min. The blank group used 70% ethanol instead of the test solution, and the control group used 70% ethanol instead of the DPPH solution. The absorbance was measured 517 nm, and they were respectively recorded as A_test_, A_blank_, and A_ctrl_, respectively.

The calculation formula is below:

$$\text{DPPH radical scavenging rate }\left(\%\right)=\left[1-\left(A_{test}-A_{ctrl}\;\right)/\;A_{blank}\right]x100\%$$

#### 2.2.3 Identification of metabolites by LC-MS

##### 2.2.3.1 Standard solutions and sample preparation

Preparation of the test solution: Firstly, 0.5 g of the sample powder (through a 40-mesh sieve) of *Esc* and its five substituents were precisely weighed in a 100 mL stoppered conical flask. Then, 80% methanol (50 mL) was added into the flask, followed by the ultrasonic treatment at room temperature for 30 min (40 kHz, 400 W). Subsequently, the weight was supplemented with 80% methanol. Next, the mixture was shaken properly. After the filtering procedure, 5 mL of the filtrate was collected and transferred to a 10 mL measuring flask. After that, 40% methanol was determined to the scale, and the mixed solution was filtered through 0.22 μm microporous membranes to obtain the test solution.

Preparation of the reference substance solution: Firstly, about 1.0 mg of each control was collected, weighed precisely, and placed in a 10 mL measuring flask. Then, the control was dissolved in methanol and diluted to the scale, followed by proper shaking to prepare a single control stock solution at a concentration of 0.1 mg/mL. Subsequently, the above stock solutions were measured, mixed with pure methanol, diluted, and finally prepared into a mixture of control solutions with a mass concentration of 1 μg/mL for each control. Finally, these control solutions were stored in the refrigerator at 4°C.

##### 2.2.3.2 Analytical conditions

Chromatographic separation was carried out on the Thermo Accucore aQ RP18 column (2.1 mm × 150 mm, 2.6 μm). The temperature of the column oven was maintained at 30°C. The mobile phase consisted of methanol (A) and 0.1% formic acid aqueous solution (B). Elution was conducted using a linear gradient of 5%–25% A for the first 12 min, 25%–30% A for 12–20 min, 30%–38% A for 20–28 min, and 38%–42% A for 28–40 min. The flow rate was set at 0.3 mL/min, and the injection volume was 3 μL.

MS experiments were performed on the Q Exactive Focus MS spectrometer equipped with a HESI interface. The ionization parameters were set as follows: spray voltage was set to 3.5 kV for negative and 3.0 kV for positive ion mode; heated capillary temperature 350°C; sheath gas at 45 arb and auxiliary gas at 15 arb; resolution of 70,000 for MS and 17,500 for MS^2^. The mass spectrometry was programmed to perform full-scan analyses over the mass range of m/z 100–1200. The collision energy of dissociation was set at 20 eV or 40 eV in the MS/MS.

##### 2.2.3.3 Metabolite analysis

Xcalibur 4.0 was employed to calculate the high resolution and accurate mass number, fit the molecular formula, and perform matching with the self-built *Erycibe* Roxb. and *Porana* plant metabolite database. The principle of 5 ppm was applied to the preliminary and rapid identification of the target metabolite, and the molecular formula of the metabolite corresponding to the chromatographic peak was inferred. The structure of the metabolite was confirmed and deduced based on the information of secondary fragments, relevant literature, and comparison with the reference substance.

#### 2.2.4 Comparative study on the efficacy of the ethanol extracts of *Esc* and its substituents

##### 2.2.4.1 Preparation of solution

Approximately 200 g of coarse powder of *Esc* and its substituents was extracted with 1.6 L of 80% ethyl alcohol by the heating circumfluence method. This procedure was repeated in triplicate, with 90 min for each. The filtrate was pooled and concentrated to obtain the extract powder, which was stored at 4°C.

##### 2.2.4.2 Cell culture and stimulation

RAW264.7 and MH7A cells were cultured in the Dulbecco’s Modified Eagle’s Medium (DMEM) containing 10% fetal bovine serum (FBS) and 1% P/S; MC3T3-E1 cells were cultured in the minimum essential medium α (MEM α) containing 10% FBS and 1% P/S. All cell lines were purchased from Procell Life Science & Technology Co., Ltd. (Wuhan, China) and incubated in a humidified atmosphere (5% CO_2_, 37°C). Subsequently, the medium was replaced with the fresh medium every 2–3 days. When the cells reached 70%–80% confluence, they were completely digested with 0.25% trypsin and then resuspended in the DMEM containing 10% FBS and 1% P/S and planted in 96-well plates (Corning, USA) at 2*10^4^ cells/200 μL, followed by the incubation at 37°C for 24 h.

#### 2.2.5 Comparison of *Esc* and its substituents attenuates the inflammatory response in LPS-Induced RAW264.7 cells

##### 2.2.5.1 Cell viability assay

Cell viability was determined by the conventional 3-(4,5-dimethylthiazol-2-yl)-2,5-diphenyl tetrazolium bromide (MTT) method. A total of 200 µL RAW264.7 cells were seeded into a 96-well plate. The following day, these cells were treated with *Esc* and its substituents at different concentrations. After the incubation for 24 h, 20 μL of MTT reagents were added to each well and incubated in darkness at 37°C for 4 h. Then, the supernatant was aspirated, and 150 µL of dimethyl sulfoxide (DMSO) was added to dissolve the purple formazan precipitates. The absorbance was measured at 490 nm using a microplate reader. The optimal stimulus concentration can be identified based on the results of this assay.

##### 2.2.5.2 Assessment of NO production

After the cells were treated and seeded in a 96-well plate according to Section 2.2.4.2, the experiment was conducted based on the control group, the model group (LPS: 0.1 μg/mL), and the LPS + *Esc*/adulterant (1, 0.75, 0.5, and 0.25 mg/mL) group. To examine the effect of *Esc* and its substituents on NO production, the amount of NO in the supernatant was detected using a commercially available NO detection kit. Briefly, equal volumes of Griess Reagent I and Griess Reagent II were added, and the absorbance was detected at 540 nm by a microplate reader. The NO content was calculated from a nitrite standard curve.

##### 2.2.5.3 Assessment of TNF-α, IL-1β, and IL-6 by ELISA Kits

The cells were treated and seeded in a 96-well plate according to Section 2.2.4.2. The culture supernatant was collected and centrifuged at 3000 rpm for 20 min. Finally, the cellular abundances of TNF-α, IL-1β, and IL-6 were detected in the medium by corresponding ELISA kit according to the manufacturer’s instructions.

##### 2.2.5.4 Comparison of the effects of *Esc* and its substituents on the proliferation and differentiation in MH7A cells

After the cells were treated and seeded in a 96-well plate according to Section 2.2.4.2. The effect of *Esc* and its substituents on the proliferation and differentiation of MH7A cells was then determined according to method 2.2.5.1.

##### 2.2.5.5 Comparison of *Esc* and its substituents attenuates LPS-Induced inhibition of osteoblast differentiation in MC3T3-E1 cells

After the cells were treated and seeded in a 96-well plate according to the procedure in Section 2.2.4.2, the experiment was conducted based on the control group, the model group (LPS: 1 μg/mL), and the LPS + *Esc*/substituent group (0.25–0.002 mg/mL). After the incubation for 24 h, these cells were treated with the MTT solution for 4 h, and blue-violet formazan crystals formed in intact cells were dissolved with DMSO. The absorbance was measured at 490 nm using a microplate reader, and EC_50_ was also calculated.

#### 2.2.6 Effect of ethanol extracts of *Esc* and *Pse* on polarization of M1/M2 macrophages

RAW264.7 cells were seeded in a 6-well plate (3*10^5^ cells/mL) at a density of 2 mL/well. After the incubation for 24 h, according to the experimental grouping, 2 mL of blank medium was added to the control group, 100 ng/mL of LPS or 10 ng/mL of IL-4 was added to the model group, the LPS or IL-4+*Esc* group (0.125, 0.0625, and 0.03125 mg/mL), and the LPS or IL-4+*Pse* group (0.125, 0.0625, and 0.03125 mg/mL). After the incubation for another 24 h, RNAs were extracted from these cells by the TRIzol reagent method. Then, cDNAs were obtained through the reverse transcription of RNA samples in each group. Subsequently, cDNAs and primers were added to the SYBR Green Master Mix system according to the manufacturer’s protocol, and the reverse transcription-quantitative polymerase chain reaction (RT-qPCR) was carried out *via* a PCR amplification apparatus. Glyceraldehyde 3-phosphate dehydrogenase (GADPH) was applied as the internal reference, and the mRNA level of iNOS, CD206, IL-6, IL-1β, TNF-α, IL-10, and Arg-1 genes were normalized with GADPH and analyzed using the 2^−ΔΔCT^ method. The gene primers are listed in Supplementary Table S1.

#### 2.2.7 Effects of ethanol extracts of *Esc* and *Pse* on osteogenic differentiation of MC3T3-E1 cells

##### 2.2.7.1 Osteoblast mineralization dyeing and ALP activity detection

After MC3T3-E1 cells were treated and incubated in 6-well plates at a density of 2*10^5^ cells/well according to the procedure in Section 2.3.2, the cells in all groups (except for the blank group) were cultured with the osteogenic induction solution (OS: MEM α supplemented with 10% FBS, 50 μg/mL ascorbic acid, 10 mM β-glycerophosphate, and 10 µM dexamethasone) to induce osteoblastic differentiation. The cells in the blank group were treated with complete MEM α. The cells in the control group were only cultured with the osteogenic differentiation medium. The cells in the experimental groups were exposed to *Esc* and *Pse* at three different concentrations (0.125, 0.0625, and 0.03125 mg/mL). After the culture for 14 days, the original culture medium was collected, and the activity of alkaline phosphatase (ALP) was measured. Specifically, the cells in the culture plate were washed twice with phosphate-buffered saline (PBS), followed by fixation in a 4% paraformaldehyde solution. After 20 min, the 4% paraformaldehyde solution was discarded and the cells were washed twice with PBS. Subsequently, the Alizarin Red S staining solution was used to stain the cells for 30 min. Then, the cells were washed with PBS and allowed to dry naturally. Next, the cells were observed and photographed using a microscope. Five images were captured per well at a magnification of about ×40 (triplicate wells per group). The mineralized nodules were quantified with the aid of the image analysis software (ImageJ, NIH, Bethesda, MA, USA), and the mineralized modules with an area exceeding 0.04 mm^2^ were counted (Liu et al., 2019; Ren et al., 2023).

##### 2.2.7.2 Determination of osteogenesis-related gene expression level changes using RT-qPCR

After these cells were treated according to the procedure described in section 2.7.1,then RNA was extracted and reverse transcribed according to method 2.6, and ALP, Runx2, Ocn, and Osx mRNA expression was detected. The gene primers are listed in Supplementary Table S1.

#### 2.2.8 Effects of ethanol extracts of *Esc* and *Pse* on apoptosis in MH7A cells

##### 2.2.8.1 Wound healing assay

MH7A cells were seeded in a 6-well plate (5*10^5^ cells/well) with a marker. After the incubation for 24 h, scratches were made using a pipette tip and rinsed twice with PBS. The experiment was conducted based on the blank group, the positive control group, and the administration group. Specifically, the blank group was supplemented with 2 mL of blank culture medium, the positive control group was supplemented with 2 mL of 0.25 μM methotrexate (MTX), and the administration group was supplemented with 2 mL of ethanol extracts from *Esc* and *Pse* at the concentration of 0.125, 0.0625, and 0.03125 mg/mL, respectively. Subsequently, the cells were placed in a 5% CO_2_ incubator at 37°C for culture. Then, the wells were photographed immediately at 0 h, 24 h, and 48 h, respectively, and the distance and area of each scratch were measured.

##### 2.2.8.2 Invasion assay

The upper surface of Transwell inserts was prepared with Matrigel (1.25 mg/mL, 20 µL/well) at 37°C for 45 min. Cell grouping and drug administration were the same as in the procedure in Section 2.5.1. After the incubation for 24 h, the noninvasive cells on the upper membrane surface were removed by wiping with a cotton swab. Then, the cells were stained with the crystal violet staining solution and photographed under a phase-contrast microscope at a magnification of about ×200. A total of five fields were photographed randomly. The experiment was repeated in triplicate.

##### 2.2.8.3 Determination of gene expression level changes using RT-qPCR

After these cells were treated according to the procedure in Section 2.5.1 and incubated for 24 h, RNAs were extracted from these cells and reversely transcribed according to the procedure in Section 2.4.1. Then, GADPH was applied as the internal reference, and the mRNA level of TNF, Casp-3, MMP-9, MMP-3, and Bcl-2 genes was normalized with GADPH and analyzed using the 2^−ΔΔCT^ method. The gene primers are listed in Supplementary Table S1.

##### 2.2.8.4 Determination of protein expression level changes using Western Blot

After these cells were treated according to the procedure in Section 2.5.1 and incubated for 24 h, the protein samples were extracted by RIPA lysates containing protease inhibitors and quantified with the Pierce BCA Protein Assay Kit. Subsequently, the proteins were separated by electrophoresis in 10% SDS-PAGE and then electro-transferred to polyvinylidene difluoride (PVDF) membranes. After being blocked with 5% non-fat milk in Tris-buffered saline with 0.1% Tween^®^ 20 detergent (TBST), the protein band was incubated with the following primary antibodies: anti-MMP-3, anti-MMP-9, anti-Caspase-3, and anti-Bcl-2. After the incubation at 4°C overnight, the PVDF membranes were incubated with the secondary antibodies at room temperature for 1 h. Finally, they were visualized using the Ultra High Sensitive ECL Kit according to the manufacturer’s instructions. Actin was used as the internal loading control.

#### 2.2.9 Statistical analyses

The related data and results of the experiment were statistically analyzed and plotted using Graphpad Prism 9.0 software. The experimental data were expressed as means ± standard deviation (SD) if they followed a normal distribution, and as M (*P25∼P75*) if they did not. If the experimental data met the normal distribution assumption, a homogeneity of variance test was conducted using one way ANOVA analysis. In case of homogeneity, intergroup comparisons were performed using the LSD method. If there was unequal variance among multiple groups of data, intergroup comparisons were made using Dunnett’s T3 method. For non-normally distributed data, nonparametric tests such as Kruskal–Wallis H method were used for intergroup comparison. A significance level of *P* < 0.05 was considered statistically significant.

## 3 Results

### 3.1 Determination of phenolic content and DPPH radical scavenging activity

**FIGURE 2:**
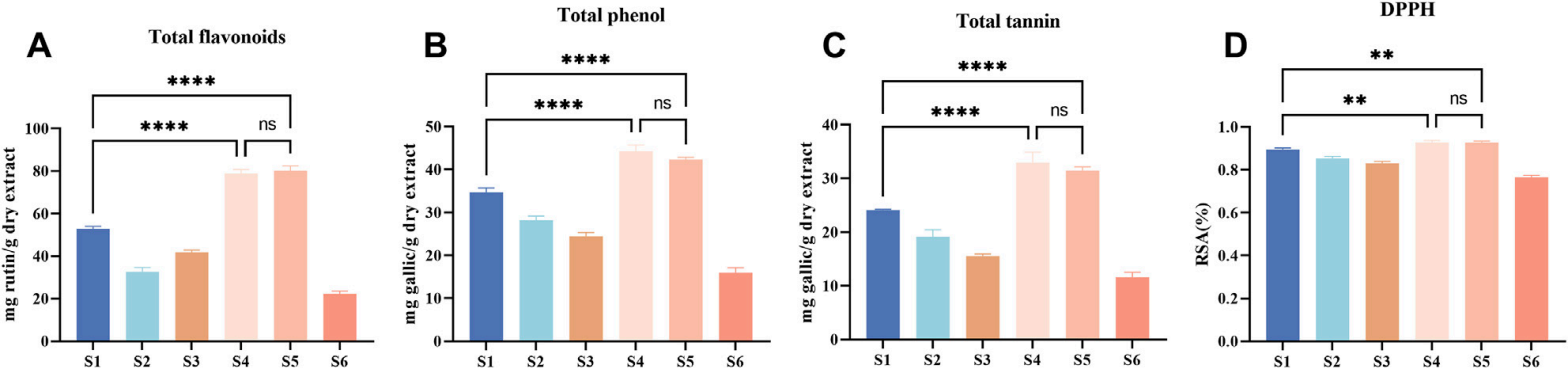
Total flavonoids, phenols and tannins, and DPPH radical scavenging activity of *Esc* (S1) and its five substituents (S2-S6). Rutin was used as the standard for total flavonoids, gallic acid was that for measurements of total phenols and total tannins. Absorbance was determined at 410 nm for total flavonoids **(A)**, 765 nm for total tannins **(B)** and total phenols **(C)**, 517 nm for DPPH **(D)**, DPPH radical scavenging activity data were calculated as the clearance rate (RSA) (%). The experimental results are presented as the average of three independent experiments (*n* = 3) and expressed as mg standard equivalent per g of dry extract. Compared with S1, ^****^ *P* < 0.0001, ^**^ *P* < 0.01. S1, *Erycibe schmidtii*; S2, *Erycibe myriantha*; S3, *Erycibe elllptilimba*; S4, *Porana sinensis*; S5, *Porana sinensis* Hemsl. Var; S6, *Porana racemosa*.

In this experiment, the phenolic substances (including total flavonoids, total phenols, and total tannins) in *Esc* and its substituents were determined and compared, as shown in Figures 2A–C. The content of total flavonoids was calculated as rutin, among which S5 had the highest content (81.88 mg/g), followed by S4 (78.76 mg/g). The other four botanical drugs can be ranked as S1, S3, S2, and S6 according to their content of total flavonoids from high to low. Besides, *Esc* and its substituents can be ranked as S4, S5, S1, S2, S3, and S6 according to their content of total phenols from high to low based on the gallic acid calculation. The content of total tannins in *Esc* and its substituents had the same trend as that of total phenols. Among them, only S4 and S5 had a higher phenolic content than *Esc* (*P* < 0.05), and there was no difference between S4 and S5. The content of other medicinal materials in substituents was lower than that in *Esc*. Similarly, the results of the DPPH free radical scavenging activity were basically consistent with those of the phenolic content, as shown in Figure 2D. Therefore, S4 and S5 may be more advantageous in replacing *Esc* in terms of the phenolic content and antioxidant activity.

### 3.2 Qualitative analysis of *Esc* and its substituents based on UPLC-Q-Exactive Focus-MS/MS

**FIGURE 3:**
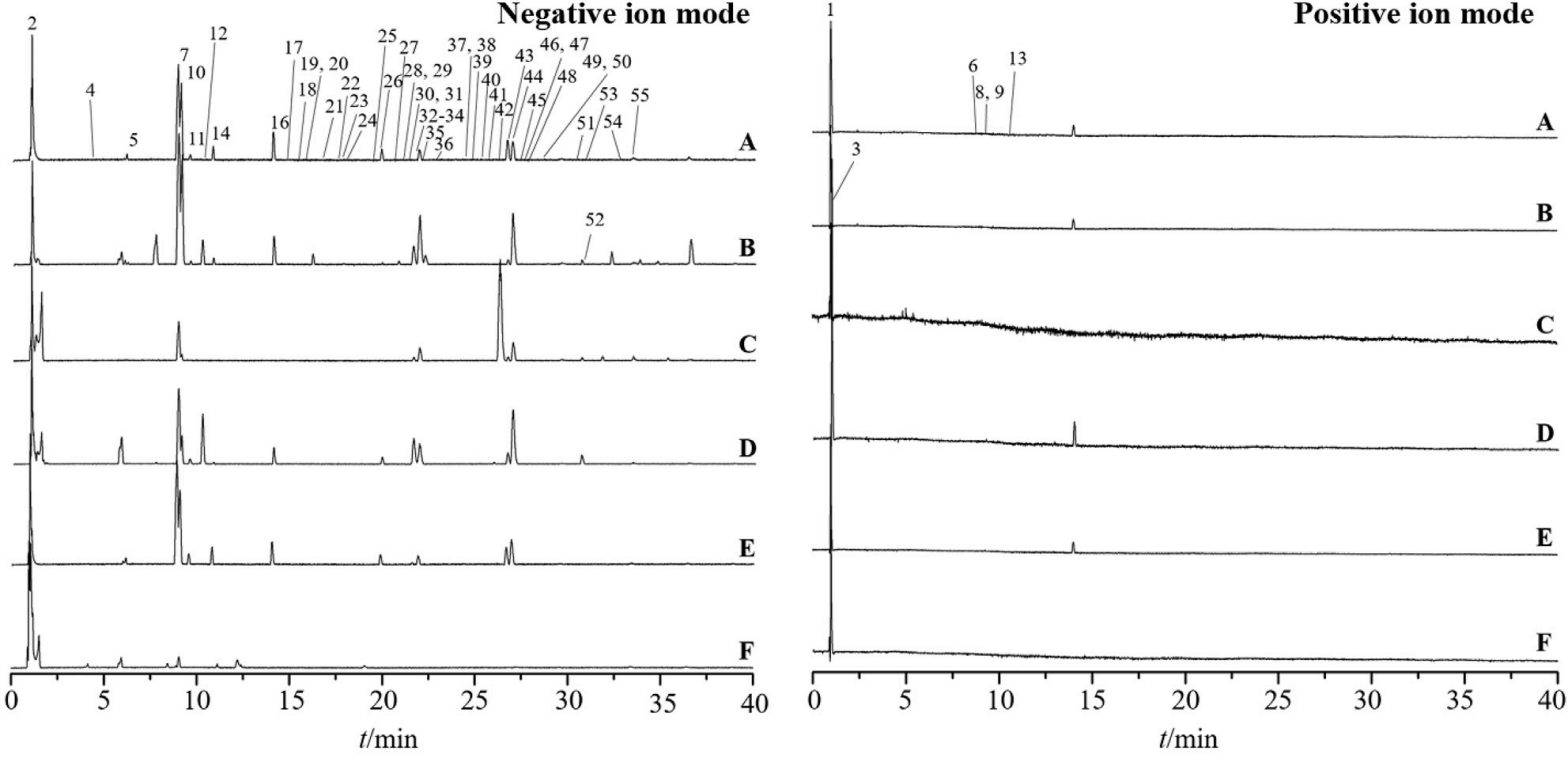
Base peak intensity chromatograms of *Erycibe schmidtii* and five potential substituents in positive and negative ion modes. *Erycibe schmidtii* **(A)**; *Erycibe myriantha* **(B)**; *Erycibe elllptilimba* **(C)**; *Porana sinensis* **(D)**; *Poranasinensis* Hemsl. Var **(E)**; *Porana racemosa* **(F)**.

The total ion chromatograms are presented in Figure 3. A total of 54 metabolites and isomers derived from *Esc* and its substituents were identified, including 28 differential metabolites. Among them, 11 metabolites were confirmed after the comparison with the reference substance. metabolites 49, 38, 39, 45, 49, and 30 were identified from *Esc*, *Erycibe myriantha* Merr., *Erycibe eliptilimba* Merr. et Chun, *Pse*, *P. sinensis* Hemsl. var. delavayi (Gagn.et Courch.) Rehd., and *P. racemosa* Roxb., respectively. Detailed information on all metabolites is listed in Supplementary Table S2. Among the 28 differential metabolites, *Esc* shared 9, 9, 15, 22, and 1 common metabolites with S2, S3, *Pse*, S5, and S6. S5 was a variant of *Pse*. Therefore, it can be concluded that *Pse* was more similar to *Esc* in metabolites, exhibiting greater potential to become its alternative.

### 3.3 Comparative efficacy of 80% ethanol extracts of *Esc* and its substituents

#### 3.3.1 Comparative effects of ethanol extracts of *Esc* and its substituents on LPS-induced inflammation in RAW264.7 cells

**FIGURE 4:**
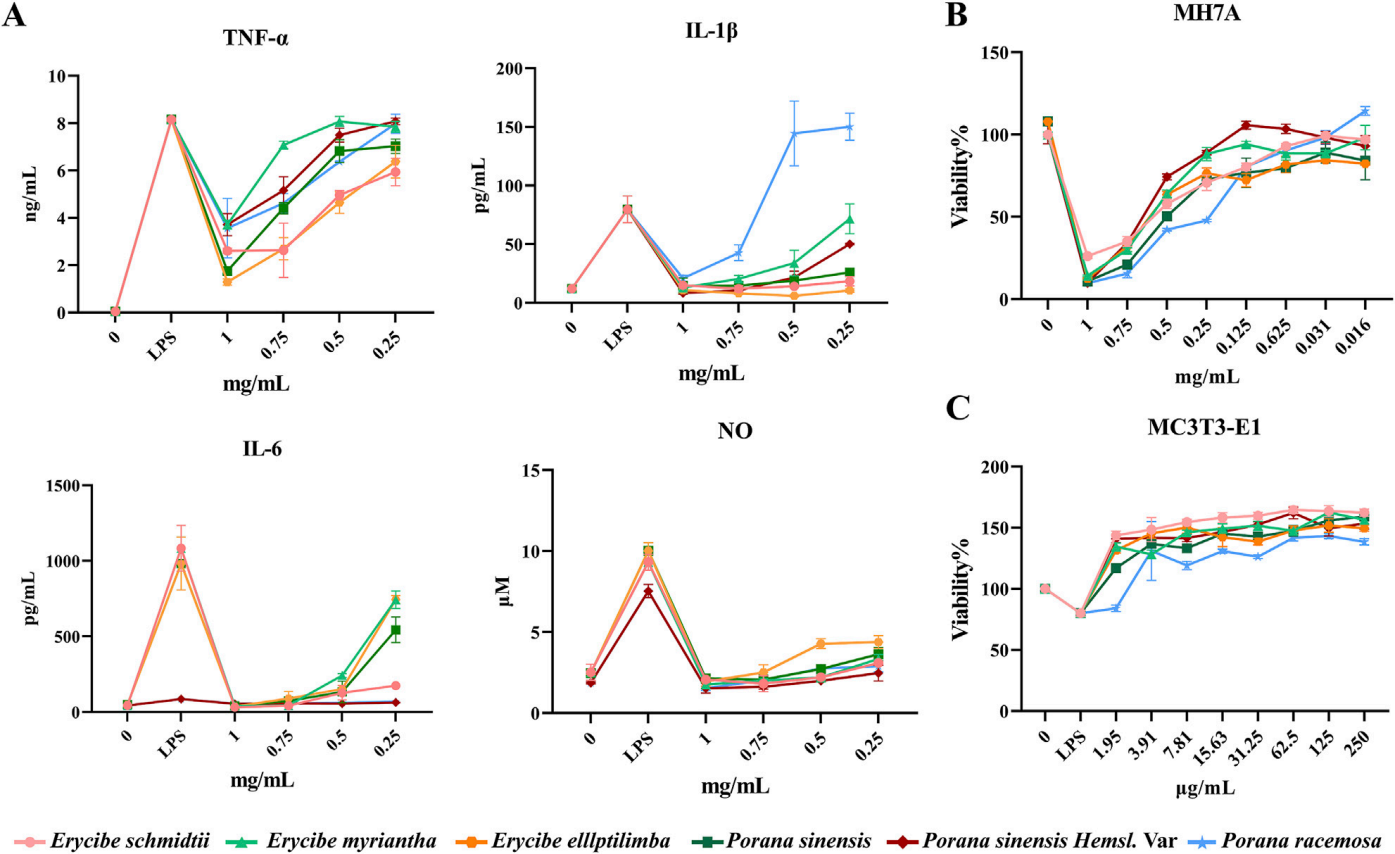
Comparative efficacy of ethanol extracts of *Esc* and its substituents *in vitro*. **(A)** The effect of *Esc* and its substituents on the pro-inflammatory factors. **(B)** The effect of *Esc* and its substituents on MH7A cells. **(C)** The effect of *Esc* and its substituents on LPS-induced MC3T3-E1 cell.

LPS-induced macrophage inflammation is a classic inflammatory model. In this study, the model was used to evaluate the anti-inflammatory effects of *Esc* and its adulterant. As shown in Figure 4A, compared with the control group, the level of NO, TNF-α, IL-1β, and IL-6 increased in the model group (LPS: 0.1 μg/mL). The expression of pro-inflammatory factors was downregulated by all drugs. However, only high concentrations of *P. racemosa* Roxb. inhibited the release of IL-1β, while low concentrations of *P. racemosa* Roxb. promoted its release. Additionally, all drug batches exhibited a weak inhibitory effect on TNF-α compared with the inhibitory effect on IL-6 and IL-1β. Therefore, it can be inferred that the anti-inflammatory effect of *Esc* and its substituents primarily involved the inhibition of such inflammatory cytokines as IL-1β and IL-6, as listed in Table 1.

**TABLE 1:** The IC_50_ value of Esc and its substituents inhibiting inflammatory factors and synoviocyte apoptosis, EC_50_ value for alleviating LPS-induced osteoblast injury ( $\bar{x}\pm s$ , *n* = 3).

IC_50_/EC_50_ (mg/mL)	*Erycibe schmidtii*	*Erycibe myriantha*	*Erycibe elllptilimba*	*Porana sinensis*	*Porana sinensis* Hemsl. Var	*Porana racemosa*
IL-6	0.072 ± 0.02	0.323 ± 0.02	0.339 ± 0.03	0.266 ± 0.06	>1	>1
TNF-α	0.549 ± 0.06	1.037 ± 0.02	0.535 ± 0.05	0.758 ± 0.03	0.937 ± 0.05	0.899 ± 0.07
IL-1β	67% ± 2%	0.615 ± 0.02	73% ± 2%	0.133 ± 0.07	0.416 ± 0.01	0.884 ± 0.02
NO	0.051 ± 0.02	0.094 ± 0.01	0.225 ± 0.04	0.094 ± 0.02	0.057 ± 0.03	0.137 ± 0.02
MC3T3-E1	0.064 ± 0.03	0.121 ± 0.01	0.180 ± 0.05	0.138 ± 0.02	0.156 ± 0.08	0.354 ± 0.13
MH7A	0.496 ± 0.05	0.513 ± 0.06	0.324 ± 0.05	0.264 ± 0.08	0.590 ± 0.05	0.290 ± 0.04

#### 3.3.2 Effects of *Esc* and its substituents on the proliferation in MH7A cells

As shown in Figure 4B, compared with the blank control group, the proliferation of MH7A cells was inhibited by all concentration treatments of *Esc* and its substituents in a dose-dependent manner. The IC_50_ results showed that the inhibitory effects of *Esc* and *Pse* on MH7A cells exhibited a higher degree of similarity, in Table 1.

#### 3.3.3 Effects of *Esc* and its substituents on osteogenic differentiation of MC3T3-E1 cells

As shown in Figure 4C, compared with the blank control group, the addition of LPS led to a decrease in cell activity, and all drugs improved LPS-induced MC3T3-E1 cell damage after administration. The EC_50_ values showed that *Esc* and *Pse* had better osteoblast proliferation-promoting activity in the inflammatory environment, in Table 1.

The results of metabolite identification and pharmacodynamic evaluation experiments demonstrated that *Pse* was more similar to *Esc* in terms of metabolites and pharmacodynamic effects, exhibiting greater potential to become an alternative to *Esc*. Based on the results of the efficacy trials, to avoid the influence of drug toxicity on the cells, 0.125, 0.0625, and 0.03125 mg/mL were selected for further experiments to reveal the acting mechanism of *Esc* and *Pse* in the treatment of RA.

### 3.4 Effects of ethanol extracts of *Esc* and *Pse* on M1/M2 polarization of macrophages

#### 3.4.1 Effects of *Esc* and *Pse* on the expression of relevant genes in RAW264.7 cells induced by LPS or IL-4

**FIGURE 5:**
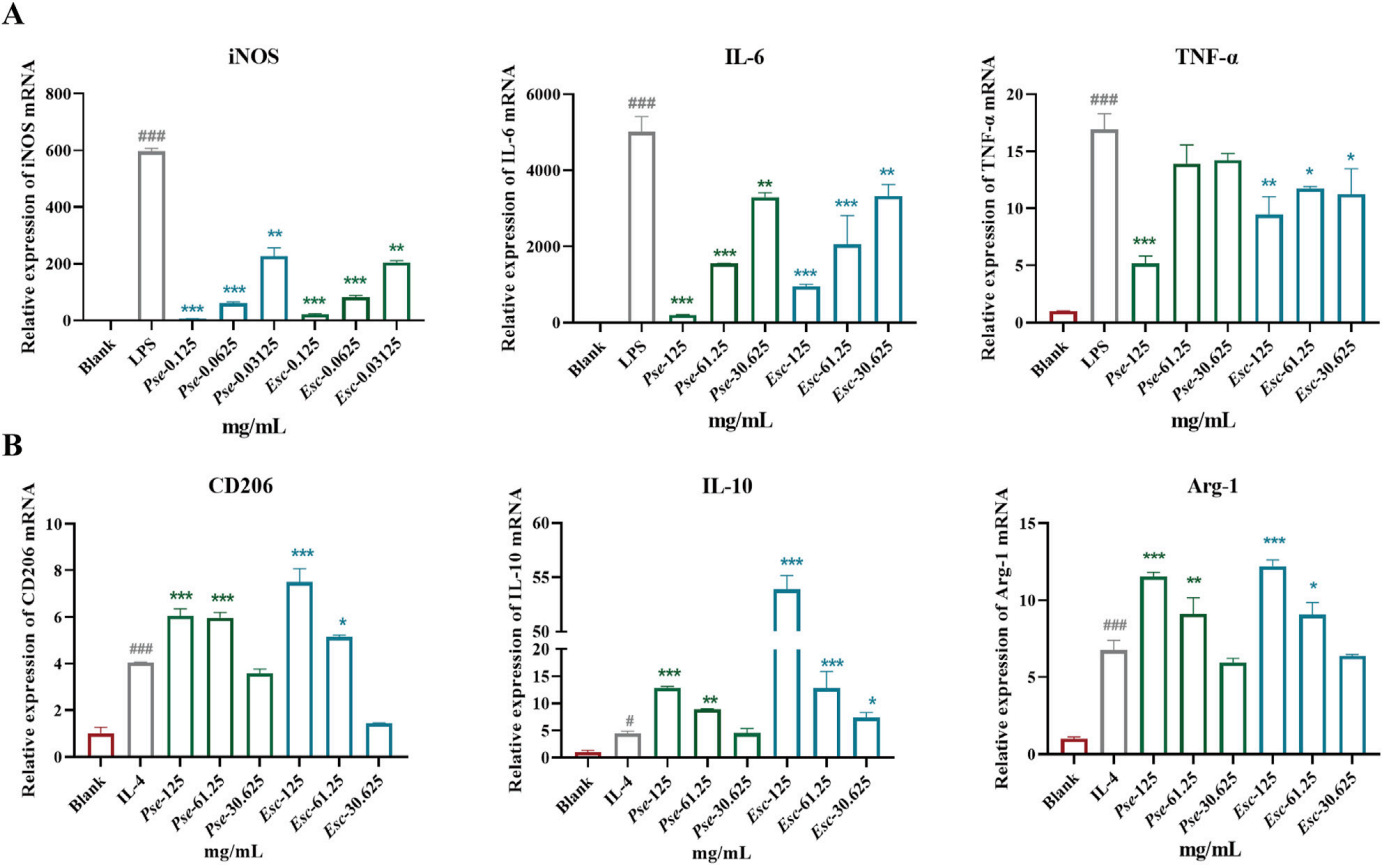
Effects of *Esc* and *Pse* on the expression of relevant genes in RAW264.7 cells induced by LPS or IL-4. **(A)** The expressions of iNOS, IL-6, and TNF-α mRNA in LPS-induced RAW264.7; **(B)** The expressions of CD206, IL-10, and Arg-1 mRNA in IL-4-induced RAW264.7. All data are presented as means ± SD. Compared to IL-4/LPS group, *p < 0.05, **p < 0.01, ***p < 0.001.

The imbalance of M1/M2 macrophages is an important factor in the pathogenesis of RA. Among them, M1 macrophages have a pro-inflammatory effect and can secrete inflammatory factors such as IL-6 and TNF-α, which can be accumulated in synovial tissues. As shown in Figure 5A, after LPS induction, the expression of iNOS, IL-6, and TNF-α was upregulated significantly, and the administration of *Esc* and *Pse* significantly inhibited the expression of these factors in a dose-dependent manner. This indicated that they could inhibit the polarization of macrophages to the M1 type. As shown in Figure 5B, after IL-4 induction, the expression of CD206, IL-10, and Arg-1 was upregulated significantly, and their expression was also upregulated after the administration of *Esc* and *Pse*. This indicated that they could promote the polarization of macrophages to the M2 type.

### 3.5 Effects of ethanol extracts of *Esc* and *Pse* on osteogenic differentiation of MC3T3-E1 cells

#### 3.5.1 *Esc* and *Pse* increased osteogenic mineralization of MC3T3-E1 cells

**FIGURE 6:**
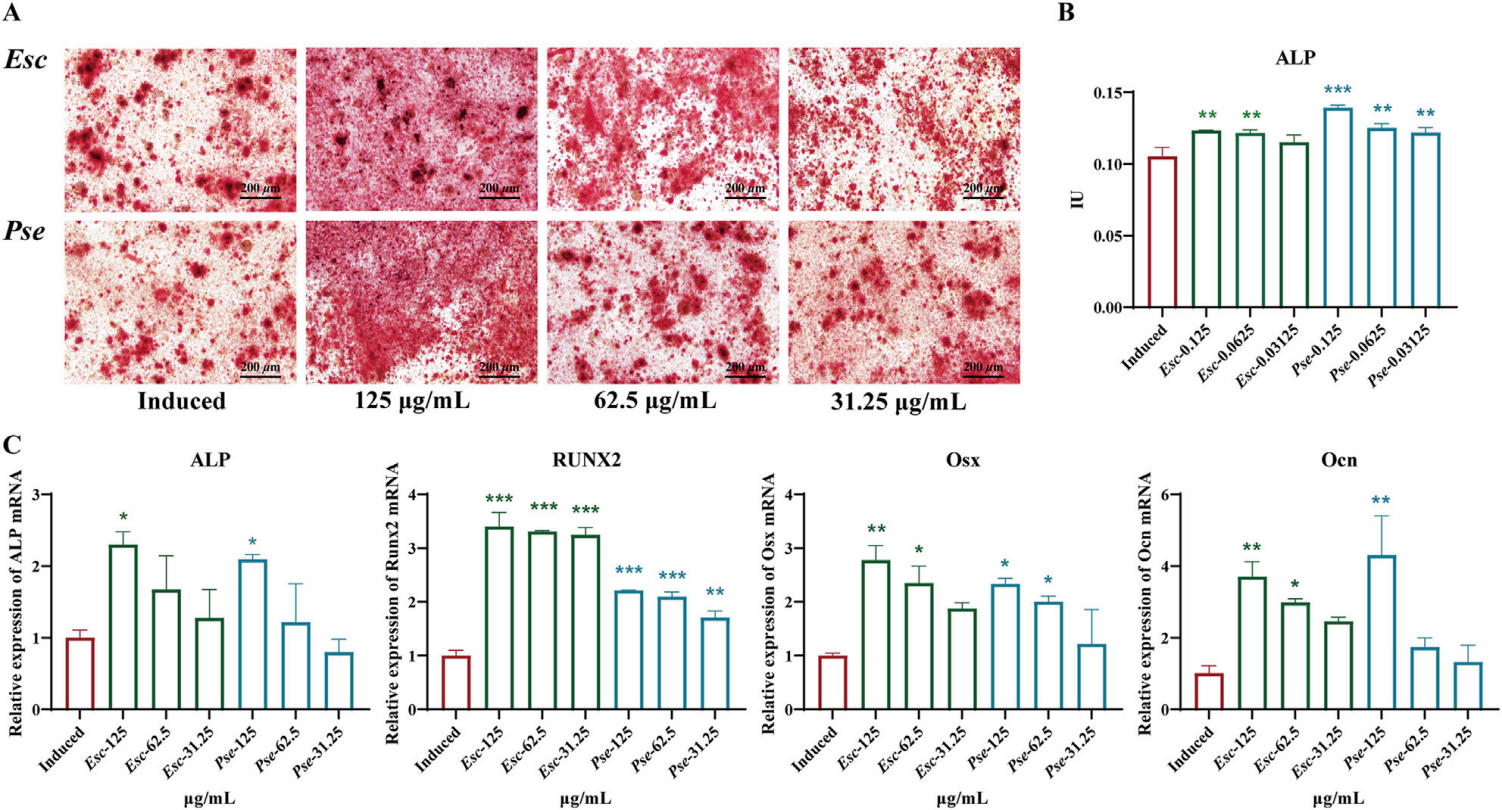
Effects of *Esc* and *Pse* on OS-induced MC3T3-E1 cells. **(A)** Mineralization of osteoblasts after 14 days of treatment with *Esc* and *Pse*; **(B)** Effects of *Esc* and *Pse* on the activity of ALP in OS-induced MC3T3-E1 cells; **(C)** Effects of *Esc* and *Pse* on the expressions of ALP, Runx2, Osx and Ocn mRNA in OS-induced MC3T3-E1 cells. All data are presented as means ± SD. Compared to induced group, *p < 0.05, **p < 0.01, ***p < 0.001.

To further validate whether *Esc* and *Pse* could control the osteogenic differentiation of MC3T3-E1 cells, ARS (Figure 6A) staining and ALP activity assays (Figure 6B) were performed. The results revealed that *Esc* and *Pse* increased the activity of ALP and the calcium deposition of MC3T3-E1 cells in a dose-response manner. When the concentration of *Esc* and *Pse* was 0.125 mg/mL, the ALP activity and calcium deposition were significantly higher compared with other concentration groups.

#### 3.5.2 *Esc* and *Pse* significantly increased osteogenic differentiation markers expression

To identify whether *Esc* and *Pse* can play a positive role in promoting the osteogenic differentiation of MC3T3-E1 cells, RT-qPCR was performed to examine the expression of osteoblast markers. As shown in Figure 6C, the mRNA expression level of Runx2, ALP, Ocn, and Osx was all upregulated significantly after the treatment with *Esc* and *Pse* compared with the control group.

### 3.6 Effects of ethanol extracts of *Esc* and *Pse* on MH7A cells

#### 3.6.1 *Esc* and *Pse* inhibits the migration in synovial fibroblasts

**FIGURE 7:**
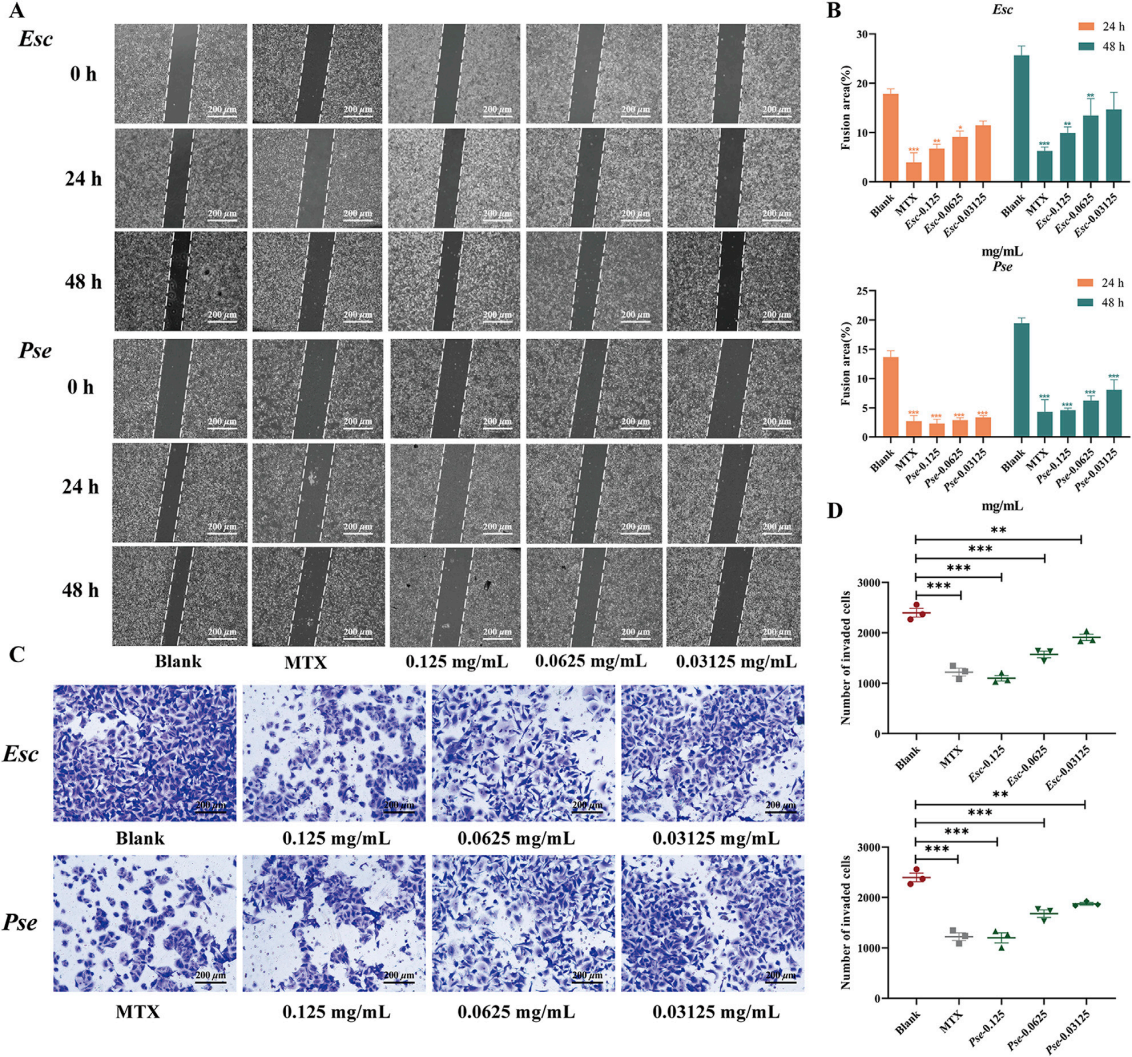
The effect of *Esc* and *Pse* on MH7A cells. **(A)** Effects of MH7A cell migration after *Esc* and *Pse* intervention. **(B)** Changes in confluent area of cells after administration of *Esc* and *Pse*; **(C)** Effects of MH7A cell invasion after *Esc* and *Pse* intervention; **(D)** The number of invasion cells after administration of *Esc* and *Pse.* All data are presented as means ± SD. Compared to Blank group, *p < 0.05, **p < 0.01, ***p < 0.001.

As shown in Figures 7A, B, compared with the blank control group, the healing ratio of cells in the positive control group decreased significantly. With an increase in the drug concentration in the administration group, the migration ability of MH7A cells was weakened, the number of cells across the scratch decreased significantly, and the percentage of migrated cells also decreased significantly, with statistical significance (*P* < 0.01). These results suggested that *Esc* and *Pse* might have the potential to inhibit cell migration in synovial fibroblasts.

#### 3.6.2 *Esc* and *Pse* inhibits the cell invasion in synovial fibroblasts

In addition to the wound-healing assay, the anti-invasive effects of *Esc* and *Pse* were also examined based on MH7A cells. According to the results in Figures 7C, D, compared with the blank control group, with an increase in the drug concentration, the invasion ability of MH7A cells was weakened significantly, and the number of cells passing through the compartment also decreased significantly, with statistical significance (*P* < 0.01). These results indicated that *Esc* and *Pse* could reduce the invasion ability of MH7A cells.

#### 3.6.3 Effect of *Esc* and *Pse* on MH7A cell apoptosis

**FIGURE 8:**
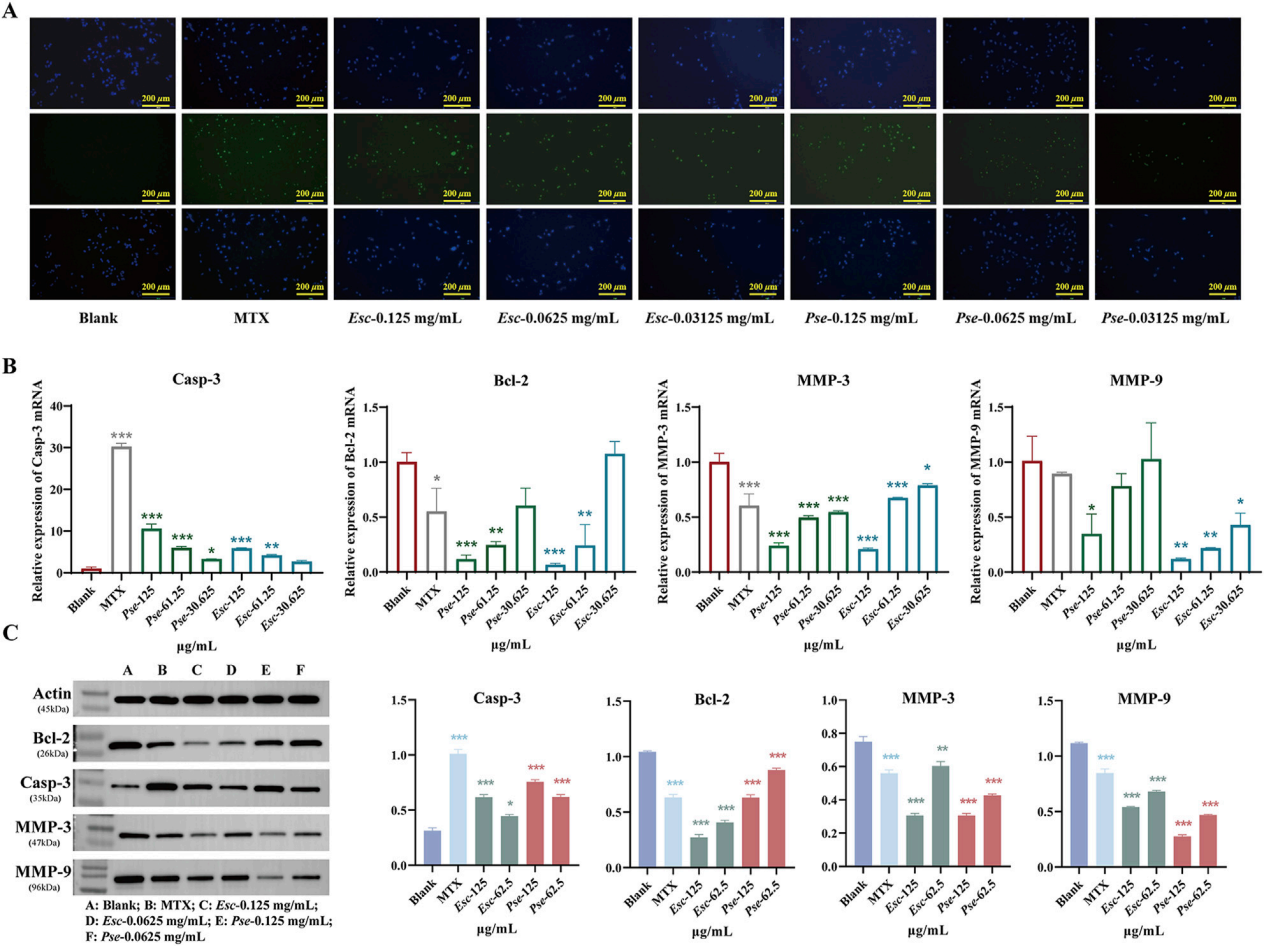
The pro-apoptosis effect of *Esc* and *Pse* on MH7A cells. **(A)** The apoptosis of MH7A cells induced by the *Esc* and *Pse* was detected by Tunel assay. **(B)** The expression of Casp-3, Bcl-2, MMP-3 and MMP-9 mRNA after administration of *Esc* and *Pse*; **(C)** The protein expression of Casp-3, Bcl-2, MMP-3 and MMP-9 after administration of *Esc* and *Pse*. All data are presented as means ± SD. Compared to Blank group, *p < 0.05, **p < 0.01, ***p < 0.001.

The TUNEL assay results showed that the fluorescence of MH7A cells increased significantly after the administration of *Esc* and *Pse*, indicating that *Esc* and *Pse* could induce the apoptosis of MH7A cells (Figure 8A). As shown in Figure 8B, compared with the blank control group, the mRNA expression level of Casp-3 genes was upregulated in each concentration group of *Esc* and *Pse*. However, the mRNA expression level of MMP-3, MMP-9, and Bcl-2 genes was downregulated in a dose-dependent manner. Consistent with the PCR results in Figure 8C, the upregulated expression of the pro-apoptotic protein Casp-3 and the downregulated expression of the anti-apoptotic protein Bcl-2 indicated that the two drugs could promote the apoptosis of MH7A cells while inhibiting the secretion of MMP-3 and MMP-9. This further confirmed that *Esc* and *Pse* could inhibit the invasion of MH7A cells.

## 4 Discussion

RA is a chronic disease characterized by persistent hyperplasia of synovial tissues, systemic inflammation, and irreversible joint damage (Gulli et al., 2020). The medicinal plant *Esc* is a widely used traditional Chinese medicine for the treatment of RA. Recently, due to over-exploitation, there has been a shortage of *Esc* resources, and more and more adulterated and fake products have emerged on the market. The proliferation of adulterated plant medicinal materials poses multi-dimensional systemic risks: in terms of quality and safety, it may introduce toxic metabolites or heavy metal contamination while causing loss of active metabolites and disruption of processing techniques; at the market level, it leads to price fluctuations of genuine products, increased quality inspection costs, and obstacles to international trade; under legal supervision, it presents challenges in accountability for counterfeiting/illegal sales and implementing quality standards; socially, it contributes to declining trust in traditional medicine and damage to biodiversity. Whether the potential substitutes can completely replace the *Esc* products is the focus of research on endangered plants, which is of great significance for the sustainable development of endangered plant resources. Given these facts, the similarity between *Esc* and its potential substitutes was evaluated in this study in terms of metabolites and antioxidant and anti-inflammatory activities. The results indicated that the changing trends of phenolic content and DPPH scavenging activity in all the medicinal materials were basically the same, and the content and antioxidant activity of *Pse* and *Psh.*V were higher than those of *Esc*. In the metabolite-based analysis, it was found that the number and types of metabolites in *Pse* were highly similar to those in *Esc*. FLSs are pivotal cellular constituents in the inflamed synovium of RA patients. The synovium comprises two layers: the intimal lining and sublayer. Under physiological conditions, the synovial lining consists of FLSs and macrophages (totaling two to three cell layers). In RA, the lining layer expands with immune cell accumulation (lymphocytes, macrophages, dendritic cells) in the sublayer. RA-FLSs develop pro-inflammatory and tissue-destructive phenotypes instead of maintaining homeostasis, upregulating inflammatory cytokines, chemokines, and matrix metalloproteinases to perpetuate inflammation and bone destruction. As FLSs play central roles in RA pathogenesis, therapeutic strategies targeting FLSs may circumvent systemic immunosuppression associated with conventional immunomodulatory therapies (Qian et al., 2024). Therefore, this study employs synovial cells, macrophages, and osteoblasts as multi-dimensional evaluation targets to assess the therapeutic effects of *Esc* and *Pse* on RA. The experiments on their anti-inflammatory effects on RAW264.7 and MC3T3-E1 cells and the anti-proliferation effects on MH7A cells showed that *Pse* was closer to *Esc* in inhibiting the release of NO and inflammatory factors, inhibiting the proliferation of synoviocytes, and alleviating the damage of osteoblasts. In summary, it can be concluded that *Pse* was the optimal potential alternative to *Esc*. Hence, *Esc* and *Pse* were selected for further investigation.

**FIGURE 9:**
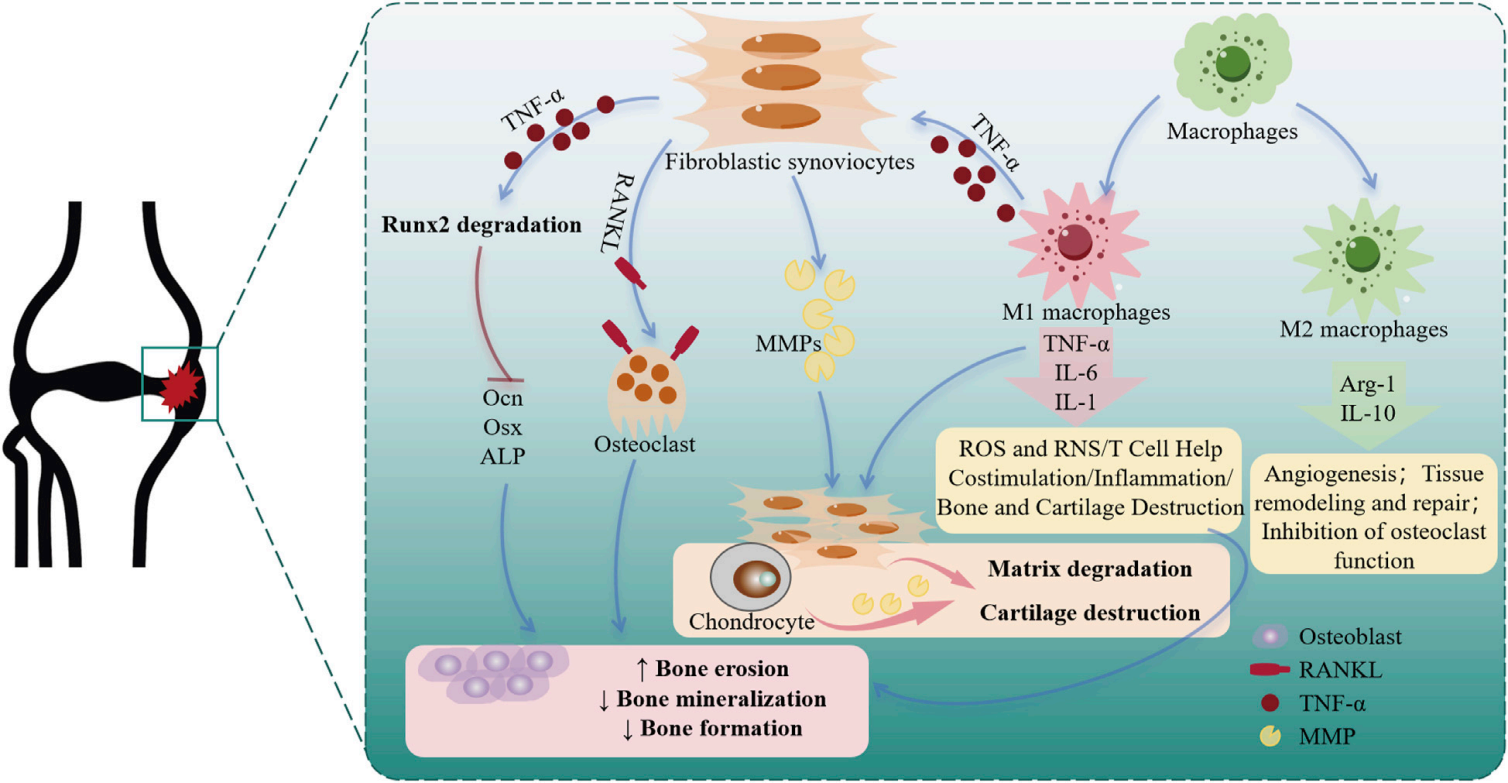
Cellular changes and roles in rheumatoid arthritis.

The pathogenesis of RA involves intricate interactions between multiple cell types, including the proliferation of synoviocytes, the mineralization of osteoblasts, and the polarization of macrophages. In this study, the above 3 cell lines were used to further explore the acting mechanism of *Esc* and *Pse*. In RA, FLSs are affected by the environment to produce MMPs, digest a variety of proteins in cartilage and supporting structures, and exhibit invasiveness. They further play a pro-inflammatory role by producing such cytokines as the activation of B cells and T cells of IL-6 and granulocyte-macrophage colony-stimulating factors (GM-CSFs) and secreting such chemokines as C-C motif chemokine ligand 2 (CCL2) and IL-8 (CXCL8). Additionally, they can recruit myeloid cells and the receptor activator of RANKL that can promote the formation of osteoclast and DickkopF-related protein 1, which can inhibit osteoblast-mediated bone repair (Qian et al., 2024). Therefore, inhibiting the proliferation of FLSs and promoting the apoptosis of FLS may present a novel therapeutic method for RA. Elevated Bcl-2 expression is associated with decreased apoptosis. In RA, FLSs have a higher proportion in the sub-synovial layer and exhibit increased Bcl-2 expression, apoptosis resistance, and abnormal proliferation. As a class of proteolytic enzymes, MMPs can promote the recruitment of inflammatory cells to the joint synovium, thereby promoting pannus formation. The overexpression of MMPs can lead to extracellular matrix degradation and articular cartilage destruction in RA rats. MMP-3 in the family can be used as a potential marker for the early diagnosis of RA with a negative anti-CCP result, and it is also an important indicator for disease evaluation, disease activity stratification, and prognosis assessment of RA (Yu et al., 2020). A detailed mechanism diagram is shown in Figure 9.

In the efficacy evaluation study, it was found that both *Esc* and *Psh* could inhibit the proliferation of MH7A cells. Besides, the further test results also showed that they could downregulated the expression of MMP-9, MMP-3, and Bcl-2, and upregulated that of Casp-3. The upregulated Casp-3 could promote the apoptosis of synoviocytes, decrease MMP-3 and MMP-9 could hinder the migration and invasion of MH7A cells. TNF-α is a cytokine that plays a central role in the inflammatory cascade that modulates the immune response. It can exert significant effects on many aspects of cellular and humoral immunity. The elevated level of TNF-α has been detected in the synovial fluid and synovium of patients with RA. Due to its influence on various cells in the synovial membrane, such as macrophages, synoviocytes, chondrocytes, and osteoclasts, TNF-α induces local inflammation and pannus formation, thus leading to cartilage erosion and bone destruction (Radner and Aletaha, 2015). TNF-α is mainly derived from activated macrophages in synovial tissues. The increased number of macrophages in the synovium is recognized as an early biomarker of RA, and the severity of joint destruction is positively correlated with the accumulation of M1 macrophages. The accumulation of M1 macrophages induces the secretion of various inflammatory factors, including IL-1β, IL-6, and TNF-α that contribute to the progression of early inflammation towards chronic arthritis. This crosstalk is mediated primarily by MHC class II and secondarily by costimulatory molecules CD80/CD86, which are overexpressed in M1 macrophages in RA. Conversely, the synovial tissue of patients with RA under remission is characterized by a higher proportion of M2 macrophages with a phagocytic activitie compared with those with active diseases (Cutolo et al., 2022; Tardito et al., 2019). Therefore, LPS and IL-4 were used to induce the polarization of macrophages to the M1/M2 type to observe the influence of *Esc* and *Pse* on the polarization of macrophages in RA. The results demonstrated that both *Esc* and *Pse* could inhibit the M1 polarization of macrophages induced by LPS and downregulate the expression of IL-6 and TNF-α. On the contrary, the addition of *Esc* and *Pse* not only promoted the expression of CD206, leading to the polarization of macrophages into the M2 phenotype, but also upregulated the expression of IL-10 and Arg-1. Hence, tissue homeostasis may be restored by regulating the balance between M1 and M2 macrophages to facilitate the anti-inflammatory effects of M2 macrophages. Based on that, it can be inferred that the ability of *Esc* and *Pse* to alleviate RA may be related to their ability to regulate the M1/M2 polarization of macrophages. In addition, the formation of a bone erosion microenvironment was observed when osteoblasts were co-cultured with RA-derived synovial tissues, which produced molecules such as inflammatory factors (TNF-α and IL-1), exosomes, and miRNAs. These factors could inhibit the proliferation and activity of osteoblasts. This can be considered one of the processes that may explain the phenomenon of bone erosion in RA (Berardi et al., 2021). In RA, osteoblast differentiation and maturation were arrested, osteoblast maturation markers (ALP and osteocalcin) were decreased, and Runx2 was degraded, a transcription factor which plays a role in osteoblast differentiation (Vimalraj, 2020; Yoon et al., 2022). Runx2 is a transcription factor that plays a role in regulating the differentiation of osteoblasts by binding to its promoter and up-regulating the expression of Ocn. The depletion of Runx2 hinders the differentiation of mesenchymal stem cells (MSCs) into pre-osteoblasts or immature osteoblasts into mature osteoblasts (Zhang et al., 2019). According to the experimental results, *Esc* and *Pse* enhanced the activity of ALP, upregulated the mRNA expression of ALP, Runx2, Osx, and Ocn, and promoted the formation of mineralized nodules, thereby promoting the ability of osteogenic mineralization.

These results suggested that *Pse* and *Esc* were not only highly similar in metabolites but also could participate in the prevention and treatment of RA at different stages based on their anti-oxidant and anti-inflammation effects, as well as their functions in anti-synovial hyperplasia and repair of osteoblast damage. This further demonstrated that *Pse* may become an alternative to *Esc*. However, the acting mechanism of the two substances in the treatment of RA was only explored *in vitro* in this study. Hence, it is necessary to further explore their bioavailability and safety based on *in vivo* animal experiments.

## 5 Conclusion

The near extinction of *Esc* plant resources results in the emergence of increasing potential substitutes in the market. Although the appearance of potential substitutes confuses the original plants of medicinal materials, it also provides ideas for expanding medicinal resources. In this study, a total of six botanical drug samples were collected from the genera *Erycibe* and *Porana*, respectively. They were evaluated at three different levels, namely, the number of metabolites, the content of phenolic substances, and the activity in *in vitro* cell experiments. Combined with the above three aspects, it was found that *Pse* of the *Porana* genus was the most similar substance to *Esc*, whose phenolic content was even higher than that of *Esc*. Besides, their acting mechanism in the treatment of RA was also explored. The results corroborated that both *Esc* and *Pse* could regulate the balance between M1 and M2 macrophages, inhibit the secretion of TNF-α, and prevent the proliferation, migration, and invasion of synoviocytes. Additionally, they could induce the apoptosis of synoviocytes by regulating Bcl-2 and Caspase-3. Meanwhile, they could increase the activity of ALP and upregulate the expression of osteogenic differentiation factors (Runx2, Osx, and Ocn), thereby promoting the mineralization of osteoblasts.

In conclusion, the potential substitute (*Pse*) with the most similar properties to *Esc* was selected in this study for the first time by comparing their metabolites and preliminary anti-oxidant and anti-inflammatory effects. Moreover, the acting mechanism of *Esc* and *Pse* in the treatment of RA was investigated by cell experiments. These findings may provide a more reliable theoretical basis for the application of *Pse* as a substitute for *Esc*. This strategy may potentially solve the supply problem of the original plants of *Esc* and could provide a foundation for the corresponding revision of the *Chinese Pharmacopoeia*, thereby contributing to the improvement of this regulatory framework. However, the acting mechanism of the two substances in the treatment of RA was only explored based on *in vitro* experiments in this study. Hence, these results need to be further verified by *in vivo* experiments. Furthermore, the safety and bioavailability of *Pse* are also worthy of further exploration.

## Data Availability

The original contributions presented in the study are included in the article/Supplementary Material, further inquiries can be directed to the corresponding authors.

## Abbreviations

ALP, alkaline phosphatase; Arg-1, Arginase-1; Bcl-2, B-cell lymphocyte/leukemia-2; CCL2, C-C Motif chemokine ligand 2DMARDs, disease-modifying anti-rheumatic drugs; DMSO, dimethylsulfoxide; EC_50_, median effective concentration; *Eob, Erycibe obtusifolia* Benth; *Esc*, *Erycibe schmidtii* Craib; FBS, fetal calf serum; FGF-2, Fibroblast Growth Factor; FLS, fibroblast-like synoviocytes; GM-CSF, Granulocyte-macrophage colony-stimulating factor; IC_50_, half maximal inhibitory concentration; IL, Interleukin; iNOS, inducible nitric oxide synthase; LPS, Lipopolysaccharide; MMP, matrix metalloproteinases; MTT, 3-(4,5-dimethylthiazol-2-yl)-2,5-diphenyl tetrazolium bromide; MTX, methotrexate; NSAIDs, non-steroidal anti-inflammatory drugs; Ocn, Osteocalcin; Osx, Osterix; *Pse*, *Porana sinensis* Hemsl.; RA, Rheumatoid arthritis; Runx2, Runt-related transcription factor 2; SD, standard deviation; TCM, Traditional Chinese medicine; TNF, tumor necrosis factor; VEGF, vascular endothelial growth factor.
