# Supplementary material for: Comparative analysis of Erycibe schmidtii Craib and its potential substitutes based on metabolites and pharmacodynamic effect
Source: Front Pharmacol. 2025 May 12;16:1510170. doi: 10.3389/fphar.2025.1510170 (PMC12104291; doi:10.3389/fphar.2025.1510170)
Supplement: Supplementary file 1 [file Table1.docx]

**Table S1** Primer sequences for the real-time PCR analysis of mRNA expression.

|  | Gene | Forward Primer (5’-3’) | Reverse Primer (5’-3’) |
| --- | --- | --- | --- |
| Raw264.7 | GAPDH | CACTCACGGCAAATTCAACGGCAC | GACTCCACGACATACTCAGCA |
|  | iNOS | GTTCTCAGCCCAACAATACAAGA | GTGGACGGGTCGATGTCAC |
|  | CD206 | GGCTGATTACGAGCAGTGGA | CATCACTCCAGGTGAACCCC |
|  | IL-6 | CCACCAAGAACGATAGTCAA | TTTCCACGATTTCCCAGA |
|  | IL-1β | AGGCTCCGAGATGAACAA | AAGGCATTAGAAACAGTCC |
|  | TNF-α | CTGTGAAGGGAATGGGTGTT | CAGGGAAGAATCTGGAAAGGTC |
|  | IL-10 | ACCTGGTAGAAGTGATGC | AAGGAGTTGTTTCCGTTA |
|  | Arg-1 | ACCCAGTCTTCGGAACCTA | TGTCCCAAAGAATGGATAGAG |
| MH7A | MMP-3 | CAGTTTGCTCAGCCTATC | CAGAGTGTCGGAGTCCAG |
|  | Casp-3 | GCTATTGTGAGGCGGTTGT | TGTTTCCCTGAGGTTTGC |
|  | TNF | CGAGTCTGGGCAGGTCTA | GTGGTGGTCTTGTTGCTTAA |
|  | MMP-9 | TCCCTGGAGACCTGAGAACC | GCCACCCGAGTGTAACCAT |
|  | Bcl-2 | ACGACCTTTAGATTCCAG | ACAGATGTCCCTACCAAC |
| MC3T3-E1 | ALP | GCACTGCCACTGCCTACT | GCTGATATGCGATGTCCTT |
|  | Runx2 | AGTCCCAACTTCCTGTGCT | GGTGAAACTCTTGCCTCGTC |
|  | Osx | GGGGAAAGGAGGCACAAA | GGAGCAAAGTCAGATGGGTAA |
|  | Ocn | AGGGAGGATCAAGTCCCG | GAACAGACTCCGGCGCTA |

**Table S2** Characterization of compounds in Esc and its adulterants by UPLC-Q-Exactive Focus-MS/MS

| No. | t_R_/min | theoretical  mass(*m*/*z*) | precusor ion | error/ppm | formula | MS2 fragments (*m*/*z*) | Compound | Source |
| --- | --- | --- | --- | --- | --- | --- | --- | --- |
| 1 | 1.01 | 144.101 9 | [M+H]^+^ | -2.77 | C_7_H_13_NO_2_ | 144.1015, 126.0910, 108.0806, 98.0962, 84.0806, 68.0696 | Baogongteng C or Erycibelline (Chen et al., 1986; Lu et al., 1986) | ABCDEF |
| 2 | 1.00 | 267.066 3 | [M-H]- | -2.24 | C16H12O4 | 267.065 7, 191.054 1, 113.023 6 | Formononetin (Matsuda et al., 2004; Morikawa et al., 2006) | ABCD |
| 2 | 1.04 | 186.112 5 | [M+H]^+^ | 1.61 | C_9_H_15_NO_3_ | 186.1128, 144.1011, 126.0905, 108.0801, 81.0694 | Baogongteng A (Yao et al., 1981) | C |
| 3 | 4.28 | 153.018 2 | [M-H]^-^ | -3.27 | C_7_H_6_O_4_ | 153.0178, 141.9110, 123.0074, 109.0278, 61.9858 | Protocatechuic acid (Song et al., 2010) | ACDEF |
| 4* | 5.8 | 353.086 8 | [M-H]^-^ | -1.42 | C_16_H_18_O_9_ | 353.0863, 191.0548, 179.0337, 173.0440, 135.0442 | Neochlorogenic acid (Zhou and Jiang, 1991) | ABCDEF |
| 5* | 8.86 | 163.038 8 | [M+H]^+^ | -1.23 | C_9_H_6_O_3_ | 163.0386, 145.0282, 135.0438, 117.0336, 89.0389 | Umbelliferone (Hu et al., 2020) | ABCDEF |
| 6* | 8.87 | 353.086 8 | [M-H]^-^ | -1.42 | C_16_H_18_O_9_ | 353.0863, 191.0560, 173.0439, 161.0223, 135.0440 | Chlorogenic acid (Song et al., 2010; Song et al., 1997b) | ABCDEF |
| 7 | 9.04 | 193.049 5 | [M+H]^+^ | -1.55 | C_10_H_8_O_4_ | 193.049 7, 178.025 8, 133.028 6, 106.064 5 | Scopoletin isomer (Song et al., 2010; Song et al., 1997a) | ABCDEF |
| 8* | 9.05 | 355.102 4 | [M+H]^+^ | -2.82 | C_16_H_18_O_9_ | 193.0493, 178.0259, 165.0543, 133.0282 | Scopolin (Song et al., 2010; Song et al., 1997b) | ABCDEF |
| 9* | 9.07 | 177.019 3 | [M-H]^-^ | 3.87 | C_9_H_6_O_4_ | 177.0182, 133.0283, 104.0253, 61.9870 | Esculetin (Hu et al., 2020) | ABCDEF |
| 10* | 9.48 | 179.033 9 | [M-H]^-^ | -2.79 | C_9_H_8_O_4_ | 179.0334, 135.0435 | Caffeic acid (Song et al., 2010) | ABCDEF |
| 11 | 10 | 211.060 1 | [M-H]^-^ | 4.26 | C_10_H_12_O_5_ | 211.0616, 196.0382, 181.0504, 163.0396, 153.0556, 116.8974, 75.0089 | Methyl 3-(2,4,5-trihydroxyphenyl) propanoate (Song et al., 2010) | ABCDE |
| 12 | 10.16 | 355.102 4 | [M+H]^+^ | -2.82 | C_16_H_18_O_9_ | 163.0389, 145.0285, 135.0444, 117.0338 | Scopolin isomer (Song et al., 2010; Song et al., 1997a) | ABCDEF |
| 13* | 10.18 | 353.086 8 | [M-H]^-^ | -1.42 | C_16_H_18_O_9_ | 353.0859, 191.0548, 173.0452, 161.0232 | Cryptochlorogenic acid (Zhou and Jiang, 1991) | ABCDEF |
| 14 | 13.19 | 367.102 4 | [M-H]^-^ | -1.91 | C_17_H_20_O_9_ | 191.0545, 173.0438, 135.0356, 111.0435, 93.0329, 87.0071 | 3-*O*-Caffeoylquinic acid methyl ester (Song et al., 2010) | ABCDEF |
| 15* | 13.99 | 193.049 5 | [M+H]^+^ | -1.55 | C_10_H_8_O_4_ | 193.0491, 178.0255, 165.0539, 149.0591, 137.0595, 133.0281 | Scopoletin (Song et al., 2010; Song et al., 1997a) | ABCDEF |
| 16 | 14.46 | 665.171 2 | [M-H]^-^ | 2.71 | C_30_H_34_O_17_ | 197.0454, 176.0113, 153.0555, 121.0293 | eryciboside Aor eryciboside B or eryciboside C (Feng, 2016; Song et al., 2010) | ABDE |
| 17 | 15.19 | 581.222 9 | [M-H]^-^ | -4.99 | C_28_H_38_O_13_ | 581.2200, 419.1674, 373.1267, 233.0796, 153.0545, 89.0537, 71.0133, 59.0134 | lyoniresinol 3*α-O-β*-D-glucopyranoside or isomer ^(Song et al., 2010)^ | ACDE |
| 18 | 15.76 | 581.222 9 | [M-H]^-^ | -4.99 | C_28_H_38_O_13_ | 581.2200, 419.1688, 373.1253, 233.0797, 153.0548, 101.0237, 59.0134 | lyoniresinol 3*α-O-β*-D-glucopyranoside or isomer (Song et al., 2010) | ACDE |
| 19 | 15.87 | 695.181 8 | [M-H]^-^ | -2.01 | C_31_H_36_O_18_ | 359.0966, 335.0766, 197.0441, 173.0437, 153.0538, 135.0438 | eryciboside F (Song et al., 2010) | ABDE |
| 20 | 16.31 | 635.160 7 | [M-H]^-^ | -1.57 | C_29_H_32_O_16_ | 191.0334, 167.0334, 121.0433 | eryciboside D or eryciboside E (Song et al., 2010) | ADE |
| 21 | 17.21 | 665.171 2 | [M-H]^-^ | 2.71 | C_30_H_34_O_17_ | 197.0455, 191.0348, 153.0556, 121.0293 | eryciboside A or eryciboside B or eryciboside C (Feng, 2016; Song et al., 2010) | ABDE |
| 22 | 17.68 | 627.192 0 | [M-H]^-^ | -4.62 | C_28_H_36_O_16_ | 627.1891, 447.1121, 429.1020, 315.0723, 263.2334, 197.0457, 183.0303, 152.0110, 109.0295 | eryciboside N or khaephuoside B or isomer (Feng et al., 2013) | AE |
| 23 | 17.86 | 635.160 7 | [M-H]^-^ | -1.57 | C_29_H_32_O_16_ | 327.3145, 191.0349, 167.0349, 123.0450 | eryciboside D or eryciboside E (Song et al., 2010) | ADE |
| 24 | 19.44 | 665.171 2 | [M-H]^-^ | 2.71 | C_30_H_34_O_17_ | 197.0455, 153.0556, 121.0292 | eryciboside A or eryciboside B or eryciboside C (Feng, 2016; Song et al., 2010) | ABDE |
| 25 | 19.87 | 533.129 0 | [M-H]^-^ | 0.938 | C_25_H_26_O_13_ | 533.1295, 371.0976, 353.0867, 335.0768, 197.0453, 173.0452, 161.0241, 153.0554, 135.0450 | 5-*O*-caffeoyl-4-*O*-syringoylquinic acid or 5-*O*-caffeoyl-3-*O*-syringoylquinic acid or 4-*O*-caffeoyl-5-*O*-syringoylquinic acid or 5-*O*-caffeoyl-4-*O*-vanilloylquinic acid methyl ester (Fan et al., 2015; Liu et al., 2014) | ABCDEF |
| 26 | 20.57 | 691.186 9 | [M-H]^-^ | 3.76 | C_32_H_36_O_17_ | 223.0609，208.0382，191.0352, 164.0478, 149.0241, 61.9883 | obtusifoside F or obtusifoside G (Liu et al., 2013) | ADE |
| 27 | 21.22 | 711.249 5 | [M-H]^-^ | -4.06 | C_33_H_44_O_17_ | 582.5746, 417.1563, 387.1094, 233.0661, 181.0504, 125.0241, 89.0243, 71.0137, 59.0137 | obtusifoside A (Liu et al., 2013) | ACDE |
| 28 | 21.28 | 691.186 9 | [M-H]^-^ | 3.76 | C_32_H_36_O_17_ | 515.1155, 497.1049, 353.0851, 317.0645, 255.0642, 191.0547, 179.0336, 161.0231, 135.0442 | obtusifoside F or obtusifoside G (Liu et al., 2013) | ABDE |
| 29 | 21.44 | 595.202 1 | [M-H]^-^ | 2.52 | C_28_H_36_O_14_ | 415.1936, 385.1287, 233.0823, 181.0506, 89.0244, 59.0139 | aketrilignoside B or isomer (Feng, 2016) | ACDE |
| 30* | 21.56 | 515.118 4 | [M-H]^-^ | -3.86 | C_25_H_24_O_12_ | 353.0851, 335.0746, 191.0547, 179.0336, 173.0441, 161.0233, 135.0442, 93.0338 | Isochlorogenic Acid B (Fan et al., 2015; Liu et al., 2014) | ABCDEF |
| 31 | 21.75 | 533.1290 | [M-H]^-^ | 0.938 | C_25_H_26_O_13_ | 371.0976, 197.0453, 173.0452, 153.0555, 121.0292 | 5-*O*-caffeoyl-4-*O*-syringoylquinic acid or 5-*O*-caffeoyl-3-*O*-syringoylquinic acid or 4-*O*-caffeoyl-5-*O*-syringoylquinic acid or 5-*O*-caffeoyl-4-*O*-vanilloylquinic acid methyl ester (Fan et al., 2015; Liu et al., 2014) | ABCDEF |
| 32* | 21.89 | 515.118 4 | [M-H]^-^ | -3.86 | C_25_H_24_O_12_ | 353.0853, 191.0548, 179.0337, 161.0233, 135.0443 | Isochlorogenic Acid A (Fan et al., 2015; Liu et al., 2014) | ABCDEF |
| 33 | 21.89 | 627.1920 | [M-H]^-^ | -4.62 | C_28_H_36_O_16_ | 167.0344, 152.0118, 123.0455, 108.0217 | eryciboside N or khaephuoside B or isomer (Feng et al., 2013) | AE |
| 34 | 22.11 | 595.202 1 | [M-H]^-^ | 2.52 | C_28_H_36_O_14_ | 415.1421, 385.1300, 233.0820, 181.0505, 89.0244, 59.0139 | Aketrilignoside B or isomer (Feng, 2016) | ACDE |
| 35 | 22.51 | 657.202 5 | [M-H]^-^ | 2.59 | C_29_H_38_O_17_ | 375.0592, 197.0457, 153.0556, 121.0294 | albibrissinoside A or 1-*O*-[6-*O*-（5-*O*-syringoyloyl-*β*-*D*-apiofuranosyl）-*β*-*D*-glucopyranosyl]-3,4,5-trimethoxybenzene (Feng et al., 2013; Liu et al., 2013) | ACE |
| 36 | 24.19 | 627.192 0 | [M-H]^-^ | -4.62 | C_28_H_36_O_16_ | 375.0695，353.0873，335.0375, 201.0169, 179.0349, 167.0348, 135.0452, 123.0451, 110.9757 | Eryciboside N or khaephuoside B or isomer (Feng et al., 2013) | AE |
| 37 | 24.34 | 657.202 5 | [M-H]^-^ | 2.59 | C_29_H_38_O_17_ | 375.0683, 197.0455, 182.0219, 153.0557, 121.0294 | Albibrissinoside A or 1-*O*-[6-*O*-（5-*O*-syringoyloyl-*β*-*D*-apiofuranosyl）-*β*-*D*-glucopyranosyl]-3,4,5-trimethoxybenzene (Feng et al., 2013; Liu et al., 2013) | ABCE |
| 38 | 24.84 | 287.055 0 | [M-H]^-^ | -0.697 | C_15_H_12_O_6_ | 243.0648, 199.0746, 177.0539, 163.0380, 137.0223, 119.0485, 93.0330, 61.9869 | Eriodictyol (Matsuda et al., 2004; Morikawa et al., 2006) | D |
| 39 | 25.47 | 282.112 5 | [M-H]^-^ | -1.06 | C_17_H_17_O_3_N | 282.1122, 197.9011, 162.0543, 145.0278, 136.0750, 119.0485 | *N-p-trans-*Coumaroyltyramine (Song et al., 2010) | ABCDEF |
| 40 | 25.9 | 559.144 6 | [M-H]^-^ | -3.56 | C_27_H_28_O_13_ | 559.1426, 353.0852, 223.0595, 191.0547, 179.0336, 164.0466, 135.0442 | 4-*O*-caffeoyl-3-*O*-sinapoylquinic acid or 5-*O*-caffeoyl-4-*O*-sinapoylquinic acid or 5-*O*-caffeoyl-3-*O*-sinapoylquinic acid (Fan et al., 2015; Liu et al., 2014) | ABCDEF |
| 41 | 26.13 | 529.134 1 | [M-H]^-^ | 4.85 | C_26_H_26_O_12_ | 367.1021, 191.0568, 173.0445, 91.0339 | methyl 3,5-dicaffeoylquinate or  methyl 3,4-dicaffeoylquinate or  methyl 4,5-dicaffeoylquinate (Fan et al., 2013) | BCDF |
| 42 | 26.64 | 533.129 0 | [M-H]^-^ | 0.938 | C_25_H_26_O_13_ | 371.0978, 197.0454, 173.0452, 155.0347, 137.0242, 93.0344 | 5-*O*-caffeoyl-4-*O*-syringoylquinic acid or 5-*O*-caffeoyl-3-*O*-syringoylquinic acid or 4-*O*-caffeoyl-5-*O*-syringoylquinic acid or 5-*O*-caffeoyl-4-*O*-vanilloylquinic acid methyl ester (Fan et al., 2015; Liu et al., 2014) | ABCDEF |
| 43* | 26.94 | 515.118 4 | [M-H]^-^ | -3.86 | C_25_H_24_O_12_ | 353.0858, 191.0548, 179.0336, 173.0442, 135.0441, 93.0339 | Isochlorogenic Acid C (Fan et al., 2015; Liu et al., 2014) | ABCDEF |
| 44 | 27.27 | 559.144 6 | [M-H]^-^ | -3.56 | C_27_H_28_O_13_ | 397.1109, 223.0595, 191.0545, 173.0439, 164.0466, 149.0231 | 4-*O*-caffeoyl-3-*O*-sinapoylquinic acid or 5-*O*-caffeoyl-4-*O*-sinapoylquinic acid or 5-*O*-caffeoyl-3-*O*-sinapoylquinic acid (Fan et al., 2013; Liu et al., 2014) | ABCDEF |
| 45 | 27.4 | 312.123 8 | [M-H]^-^ | 0 | C_18_H_19_O_4_N | 312.1238, 297.1001, 190.0508, 178.0506, 148.0528, 135.0451 | N-*trans*-Feruloyltyramine or N-cis-Feruloyltyramine (Song et al., 2010) | ABCDEF |
| 46 | 27.47 | 529.134 1 | [M-H]^-^ | 4.85 | C_26_H_26_O_12_ | 374.4986, 191.0595, 173.0441, 91.0339 | methyl 3,5-dicaffeoylquinate or  methyl 3,4-dicaffeoylquinate or  methyl 4,5-dicaffeoylquinate (Fan et al., 2013) | BCDF |
| 47 | 27.87 | 683.218 2 | [M-H]^-^ | -1.98 | C_31_H_40_O_17_ | 223.0613, 205.0504, 179.0713, 164.0482, 149.0244 | 1-*O*-[6-*O*-（5-*O*-sinapoyl-*β*-*D*-apiofuranosyl）-*β*-D-glucopyranosyl]-3,4,5-trimethoxyphenol or isomer (Feng et al., 2014) | ABE |
| 48 | 28.66 | 337.107 1 | [M-H]^-^ | -2.67 | C_20_H_18_O_5_ | 337.1062, 191.0548, 173.0344, 119.0494, 93.0339 | Erythrinin B (Matsuda et al., 2004; Morikawa et al., 2006) | ABCDEF |
| 49 | 28.83 | 683.218 2 | [M-H]^-^ | -1.98 | C_31_H_40_O_17_ | 553.0609, 375.0712, 296.1151, 223.0614, 205.0505, 179.0714, 164.0476, 149.0244 | 1-*O*-[6-*O*-（5-*O*-sinapoyl-*β*-*D*-apiofuranosyl）-*β*-D-glucopyranosyl]-3,4,5-trimethoxyphenol or isomer (Feng et al., 2014) | ABE |
| 50 | 30.66 | 559.144 6 | [M-H]^-^ | -3.56 | C_27_H_28_O_13_ | 397.1108, 353.0849, 223.0595, 191.0547, 173.0441, 161.0231, 143.0339, 135.0442 | 4-*O*-caffeoyl-3-*O*-sinapoylquinic acid or 5-*O*-caffeoyl-4-*O*-sinapoylquinic acid or 5-*O*-caffeoyl-3-*O*-sinapoylquinic acid (Fan et al., 2013; Liu et al., 2014) | ABCDEF |
| 51 | 31.02 | 529.134 1 | [M-H]^-^ | 4.85 | C_26_H_26_O_12_ | 374.4986, 191.0595, 173.0441, 91.0339 | methyl 3,5-dicaffeoylquinate or  methyl 3,4-dicaffeoylquinate or  methyl 4,5-dicaffeoylquinate (Fan et al., 2013) | BCDF |
| 52 | 31.04 | 701.301 5 | [M-H]^-^ | 2.89 | C_33_H_50_O_16_ | 603.1063, 304.1004, 197.0456, 179.0344, 161.0242, 153.0554, 121.0295 | Eryciboside I (Song et al., 2010) | AE |
| 53 | 33.07 | 515.118 4 | [M-H]^-^ | -3.86 | C_25_H_24_O_12_ | 353.0852, 191.0548, 179.0337, 173.0442, 135.0442 | Cynarin (Zhou and Jiang, 1991) | ABCDEF |
| 54 | 33.99 | 533.129 0 | [M-H]^-^ | 0.938 | C_25_H_26_O_13_ | 371.0979, 197.0451, 173.0453, 155.0340, 93.0349 | 5-*O*-caffeoyl-4-*O*-syringoylquinic acid or 5-*O*-caffeoyl-3-*O*-syringoylquinic acid or 4-*O*-caffeoyl-5-*O*-syringoylquinic acid or 5-*O*-caffeoyl-4-*O*-vanilloylquinic acid methyl ester (Fan et al., 2015; Liu et al., 2014) | ABCDEF |

* Confirmation by comparison of the reference; Source of medicinal materials: A-*Erycibe schmidtii,* B-*Erycibe myriantha*, C-*Erycibe elllptilimba*, D-*Porana sinensis*, E-*Porana sinensis* Hemsl. Var, F-*Porana racemosa*.

**Reference**

Chen, Z., Xu, P., Yao, T., 1986. Chemical study of *Erycibe obtusifolia* Benth. -- III. Identification of bao gong teng B and study of bao gong teng C. Chinese Herbal Medicines 17, 2-3.

Fan, L., Wang, Y., Liang, N., Huang, X.J., Fan, C.-l., Wu, Z.L., He, Z., Li, Y.-l., Ye, W.C., 2015. Quinic acid derivatives and coumarin glycoside from the roots and stems of Erycibe obtusifolia. Phytochemistry Letters 14, 185-189.

Fan, L., Wang, Y., Liang, N., Huang, X.J., Li, M.M., Fan, C.L., Wu, Z.L., Li, Y.L., Ye, W.C., 2013. Chemical constituents from the roots and stems of Erycibe obtusifolia and their in vitro antiviral activity. Planta Med 79, 1558-1564.

Feng, Z.-m., Song, S., An, Y.-W., Yang, Y., Jiang, J.-S., Zhang, P., 2014. Hepatoprotective acyl glycosides obtained from Erycibe hainanesis. Phytochemistry Letters 9, 163-167.

Feng, Z.-M., Zhao-ZhenYang, Peng-FeiYang, Ya-NanJiang, Jian-shuangZhang, Pei-cheng, 2016. Two new quinic acid derivatives and one new lignan glycoside from Erycibe obtusifolia. Phytochemistry Letters 17.

Feng, Z.M., Song, S., He, J., Yang, Y.N., Jiang, J.S., Zhang, P.C., 2013. Acyl glycosides with rare β-D-apiofuranosyl-β-D-glucopyranosyl-β-D-apiofuranosyl from Erycibe hainanesis. Carbohydr Res 380, 59-63.

Hu, J., Yang, Y., Ren, H., Cui, X., Liu, X., Luo, J., Chen, Z., 2020. Identification of Chemical Constituents in Caulis of Erycibe schmidtii byUPLC-Q-Exactive Focus-MS/MS. Chinese Journal of Experimental Traditional Medical Formulae 26, 124-132.

Liu, Z., Feng, Z., Yang, Y., Jiang, J., Zhang, P., 2014. Acyl quinic acid derivatives from the stems of Erycibe obtusifolia. Fitoterapia 99, 109-116.

Liu, Z.Z., Zhan, Z.L., Liu, F., Yang, Y.N., Feng, Z.M., Jiang, J.S., Zhang, P.C., 2013. Acyl glycosides lignans, coumarins, and terpenes from the stems of Erycibe obtusifolia. Carbohydr Res 372, 47-54.

Lu, Y., Yao, T., Chen, Z., 1986. Study on the chemical constituents of *Erycibe elliptilimba* Merr. & Chun. Acta Pharmaceutica Sinica, 829-835.

Matsuda, H., Morikawa, T., Xu, F., Ninomiya, K., Yoshikawa, M., 2004. New isoflavones and pterocarpane with hepatoprotective activity from the stems of Erycibe expansa. Planta Med 70, 1201-1209.

Morikawa, T., Xu, F., Matsuda, H., Yoshikawa, M., 2006. Structures of new flavonoids, erycibenins D, E, and F, and NO production inhibitors from Erycibe expansa originating in Thailand. Chem Pharm Bull (Tokyo) 54, 1530-1534.

Song, S., Li, Y., Feng, Z., Jiang, J., Zhang, P., 2010. Hepatoprotective constituents from the roots and stems of Erycibe hainanesis. J Nat Prod 73, 177-184.

Song, W., Jin, R., Liu, J., 1997a. Study on the chemical constituents of *Erycibe schmidtii* Craib. China Journal of Chinese Materia Medica, 39-40+64.

Song, W., Liu, J., Jin, R., 1997b. [Chemical constituents of the stems of Erycibe schmidtii Craib]. Zhongguo Zhong Yao Za Zhi 22, 359-360, 384.

Yao, T., Chen, Z., Yi, D., Xu, G., 1981. Study on the chemical constituents of *Erycibe obtusifolia* Benth.——Ⅱ.New mydriatic——Structure of Bao gong teng A. Acta Pharmaceutica Sinica, 582-588.

Zhou, Y., Jiang, J., 1991. Overview of pharmacological research of Rhubarb. Pharmacology and Clinics of Chinese Materia Medica, 41-48.
