# Supplementary material for: Comparative analysis of Erycibe schmidtii Craib and its potential substitutes based on metabolites and pharmacodynamic effect
Source: Front Pharmacol. 2025 May 12;16:1510170. doi: 10.3389/fphar.2025.1510170 (PMC12104291; doi:10.3389/fphar.2025.1510170)
Supplement: Supplementary file 2 [file DataSheet1.docx]

**Actin**










**Bcl-2**










**Casp-3**










**MMP-3**










**MMP-9**
